# Genetic Deficiency of p53 Leads to Structural, Functional, and Synaptic Deficits in Primary Somatosensory Cortical Neurons of Adult Mice

**DOI:** 10.3389/fnmol.2022.871974

**Published:** 2022-04-07

**Authors:** Haixia Kuang, Tao Liu, Cui Jiao, Jianmei Wang, Shinan Wu, Jing Wu, Sicong Peng, Andrew M. Davidson, Shelya X. Zeng, Hua Lu, Ricardo Mostany

**Affiliations:** ^1^Department of Pediatrics, The First Affiliated Hospital of Nanchang University, Nanchang, China; ^2^Department of Biochemistry and Molecular Biology, Tulane University School of Medicine, New Orleans, LA, United States; ^3^Department of Pharmacology, Tulane University School of Medicine, New Orleans, LA, United States; ^4^Tulane Brain Institute, Tulane University, New Orleans, LA, United States

**Keywords:** p53, pyramidal neuron, barrel cortex, dendritic spine, patch-clamp technique, two-photo imaging

## Abstract

The tumor suppressor p53 plays a crucial role in embryonic neuron development and neurite growth, and its involvement in neuronal homeostasis has been proposed. To better understand how the lack of the *p53* gene function affects neuronal activity, spine development, and plasticity, we examined the electrophysiological and morphological properties of layer 5 (L5) pyramidal neurons in the primary somatosensory cortex barrel field (S1BF) by using *in vitro* whole-cell patch clamp and *in vivo* two-photon imaging techniques in p53 knockout (KO) mice. We found that the spiking frequency, excitatory inputs, and sag ratio were decreased in L5 pyramidal neurons of p53KO mice. In addition, both *in vitro* and *in vivo* morphological analyses demonstrated that dendritic spine density in the apical tuft is decreased in L5 pyramidal neurons of p53KO mice. Furthermore, chronic imaging showed that p53 deletion decreased dendritic spine turnover in steady-state conditions, and prevented the increase in spine turnover associated with whisker stimulation seen in wildtype mice. In addition, the sensitivity of whisker-dependent texture discrimination was impaired in p53KO mice compared with wildtype controls. Together, these results suggest that p53 plays an important role in regulating synaptic plasticity by reducing neuronal excitability and the number of excitatory synapses in S1BF.

## Introduction

Since its discovery in 1979 (Lane and Crawford, 1979; Linzer and Levine, 1979), p53 has been considered as the “cellular gatekeeper for growth and division” (Vogelstein et al., 2000), because it can activate or repress genes crucial for the regulation of cell death, growth, and proliferation. For this reason, its role in cancer has been extensively studied (Kastenhuber and Lowe, 2017; Lu, 2019; Zhou et al., 2019; Levine, 2020). In addition to exerting tumor-suppressive effects, p53 has also been shown to play a physiological role in specific organ functions, such as maternal reproduction (Levine et al., 2011) and central nervous system (CNS) development (Jacobs et al., 2006) and function (Jewett et al., 2015). For instance, p53 knockout (KO) mice display many abnormalities, including that a fraction of p53KO embryos present exencephaly, i.e., the lack of meninges and skull (Armstrong et al., 1995; Sah et al., 1995). Furthermore, the involvement of p53 in homeostatic plasticity mechanisms has been previously proposed (Jewett et al., 2015; Lee et al., 2018). Young p53KO mice seem to develop normally and without readily detected physiological defects, until they start developing tumors at 3 months of age (Donehower et al., 1992), likely due to the compensatory role of the other proteins of the p53 family such as p63 or p73 (Agostini et al., 2018). However, a number of studies have shown that p53KO mice suffer from multiple neurological problems, including learning deficiencies, increased thigmotaxis, and walking synchronization deficits (Amson et al., 2000; Campana et al., 2003). These abnormalities may result from the role of p53 in regulating neurite outgrowth, axon regeneration, and neuronal homeostasis, although this needs to be further validated. After facial nerve axotomy, the number of regenerating fibers in p53-null mice was reduced compared with wildtype (WT) mice (Di Giovanni et al., 2006), and consistent with this finding, pharmacological inhibition of p53 in cultured hippocampal neurons induced axonal growth cone collapse, whereas overexpression of p53 resulted in larger axonal growth cones (Qin et al., 2009). Additionally, p53 was associated with homeostatic control of neuronal activity *via* ubiquitination and downregulation of the glutamate receptor subunit 1 (GluA1) of the AMPA receptor (Jewett et al., 2015). Moreover, p53 activation has been shown to increase spine formation in cultured hippocampal neurons treated with erythropoietin (EPO) and carbamylated EPO (cEPO; Choi et al., 2014). However, little is known about the role that p53 plays in the function and synaptic plasticity of principal neurons, and whether its role may contribute to sensory information processing.

In our attempt to address this question, we generated a p53KO mouse line that expressed GFP in a subset of layer (L) 5 projection pyramidal neurons and examined gross and fine structural changes in anatomy, performed *in vitro* electrophysiological recordings, and *in vivo* two-photon excitation imaging of L5 pyramidal neurons in the primary somatosensory cortex barrel field (S1BF). We also tested whether sensory processing was affected by the p53 deficiency using a whisker-dependent texture discrimination task. As a result, we found that the deficiency of p53 profoundly alters both electrophysiological and morphological properties of L5 pyramidal neurons in S1BF of p53KO mice. Interestingly, the dendritic spine turnover in response to whisker stimulation was also impaired in the p53KO mice. Furthermore, these mice displayed an impaired performance in the whisker-dependent task compared to WT mice. These results unveil the unknown role of p53 in neuronal function and experience-dependent modification of sensory circuits.

## Materials and Methods

### Animals

Thy1-eGFP-M (GFP-M; The Jackson Laboratory, Bar Harbor, ME, USA; RRID:IMSR_JAX:007788; Feng et al., 2000), p53^+/+^ (WT), and p53^−/−^ (KO, p53KO; Zhang et al., 2017) mice with C57BL/6 genetic background were used in the present study. To obtain GFP-M:p53KO mice for the *in vivo* imaging experiments, GFP-M mice were first crossed with p53^−/−^ mice, then the double GFP-M:p53 heterozygous offspring was further intercrossed to generate p53^+/+^:GFP-M and p53^−/−^:GFP-M mice. The Thy1-eGPF-M mouse line primarily labels projection neurons (Feng et al., 2000). The mice genotypes were determined by PCR analysis of the genomic DNA extracted from tail snips. Both male and female mice aged 3–5 months were used for *in vivo* two-photon excitation (2PE) imaging experiments. Male mice aged 2–3 months were used for the rest of the experiments. The mice were housed in temperature-controlled facilities, maintained at 24 ± 1°C and 50%–60% humidity, with a 12:12-h light/dark cycle. Food and water were available *ad libitum*. Given that p53KO mice develop tumors mostly between 4–5 months of age (Jacks et al., 1994), we did not use mice older than 5 months old in this study. All mice used were examined to ensure no tumor presence at the time of the experiment. Experiments were conducted in accordance with the National Institutes Health “Guide for the Care and Use of Laboratory Animals” and were approved by the Institutional Animal Care and Use Committees at Tulane University School of Medicine and Nanchang University.

### Nissl Cresyl Violet Staining

Six p53^+/+^ mice and six p53^−/−^ mice were deeply anesthetized with urethane (1.5 g/kg; i.p.) and transcardially perfused with 30 ml of ice-cold normal saline. Brains were removed, weighed, and immersed in 10% formalin for fixation for at least 24 h. For Nissl staining, six consecutive coronal paraffin-embedded brain sections (3 μm) were stained with cresyl violet (G1430; Solarbio, Beijing, China) and imaged using a biological navigator (FSX100; Olympus, Tokyo, Japan). The cortex thickness and the number of neurons were measured using the *Cell Counter* tool in ImageJ software (version 1.50i, RRID: SCR_003070^1^) within three random regions of interest (200 μm × 150 μm) in L5 of S1BF. The average number of neurons from three to five sections was taken as the final value for each mouse.

### Acute Brain Slice Preparation

Acute coronal brain slices for electrophysiology were prepared as described in our previous study (Wu et al., 2021). Mice were deeply anesthetized with urethane and transcardially perfused with 20 ml ice-cold carbonated (95% O_2_ and 5% CO_2_) dissection solution containing (mM): 240 sucrose, 2.5 KCl, 3.5 MgCl_2_, 0.5 CaCl_2_, 1.25 NaH_2_PO_4_, 0.4 ascorbic acid, 2 sodium pyruvate, 25 NaHCO_3_, and 1 kynurenic acid. Brains were quickly removed after decapitation and put into a chilled dissection solution. Coronal slices containing S1BF (350 μm thick) were obtained using a Leica VT1000S (Wetzlar, Germany) vibratome and then transferred to oxygenated artificial cerebrospinal fluid (aCSF) containing (mM): 117 NaCl, 3.6 KCl, 1.2 NaH_2_PO_4_, 2.5 CaCl_2_, 1.2 MgCl_2_, 25 NaHCO_3_, 11 D-glucose, and 2 sodium pyruvate for 30 min at 32°C to recovery. Afterward, slices were maintained for at least 1 h at room temperature (RT, 22–24°C) in aCSF prior to being moved to the recording chamber.

### *In vitro* Electrophysiological Recordings

Whole-cell voltage- and current-clamp recordings were obtained as previously described (Popescu et al., 2017; Wu et al., 2018). In brief, one brain slice was moved to the recording chamber and continuously perfused with aCSF at RT with a perfusion rate of 2 ml/min. Recording electrodes were pulled from borosilicate glass (1.5 mm OD, 1.12 mm ID; World Precision Instruments, Shanghai, China) with a micropipette puller (P-97; Sutter Instrument, Novato, CA, USA). The typical resistance of the pipette was 3–6 MΩ when filled with intracellular solution containing (mM): 130 K-gluconate, 5 KCl, 10 Na_2_-phosphocreatine, 0.5 EGTA, 10 HEPES, 4 Mg-ATP, and 0.3 Li-GTP (pH = 7.3 adjusted with KOH, 295 mOsm). The barrel field was visualized at low magnification under bright-field illumination and a target region in L5 directly below an L4 barrel was selected for recording. Individual L5 pyramidal neurons were visually identified at 40× magnification using an infrared differential interference contrast camera (IR-1000; Dage-MTI, Michigan City, IN, USA) with an upright microscope (BX51WI; Olympus). Signals were amplified with an EPC-10 amplifier and Patchmaster software (RRID: SCR_000034; HEKA Electronik, Reutlingen, Germany). The series resistances were typically 10–30 MΩ and were monitored throughout the recording period. Data were excluded if the series resistance changed by >20%. To assure the clear identification of each neuron in the anatomy *post hoc* analysis, only one or two cells were patched in each slice. Only well-labeled neurons with stable resting membrane potentials (V_rest_ ≤ −50 mV) were included in the final analysis. Data were not adjusted for a liquid junction potential of −15 mV. All the reagents used for the electrophysiological experiment were obtained from Sigma-Aldrich (St. Louis, MO, USA) unless otherwise mentioned.

### Electrophysiology Data Analysis

Three major parameters of L5 pyramidal neurons were analyzed here, passive intrinsic properties, active intrinsic properties by intracellular current injection, and miniature excitatory postsynaptic currents (mEPSCs). Membrane resting potential (V_rest_; in mV) was defined as the membrane potential within 30 s after establishing the whole cell configuration with no current applied (*I* = 0 pA). Input resistance (R_in_; in MΩ) was measured from the current response to a 10-mV hyperpolarizing step in voltage clamp. Active intrinsic properties were examined by injecting a series of depolarizing and hyperpolarizing current steps (2 s duration, 20 pA increments). Rheobase current (in pA) was determined as the smallest current step to elicit at least one action potential (AP). AP threshold (AP_thre_; in mV) was defined as the inflection point during spike initiation of the first AP. AP amplitude (AP_amp_; in mV) was defined as the difference between the firing threshold and its maximum positive peak. AP half-width (HW; in ms) was calculated at 50% of AP amplitude. To investigate the sag, neurons were given a series of 2 s hyperpolarized current pulses from V_rest_. The sag ratio was calculated and expressed as a percentage by dividing the steady-state potential by the peak potential at −280 pA. Miniature EPSCs (in pA) were recorded at a holding potential of −70 mV in voltage clamp after 10-min application of 0.5 μM tetrodotoxin (TTX; Tocris, Bristol, UK). Membrane properties were analyzed using Clampfit 10.7 (pClamp, RRID: SCR_011323; Molecular devices, Hercules, CA, USA). Amplitudes and frequencies of mEPSCs were analyzed using the MiniAnalysisProgram (RRID: SCR_002184; Synaptosoft).

### Western Blot

The S1BF of the cerebral cortex from WT and p53KO mice was dissected in a cold RIPA buffer. For Western blot assays, samples (200 μg protein for GluA1 and 40 μg protein for β-actin) were transferred onto a polyvinylidene fluoride (PVDF) membrane (Bio-Rad Laboratories, Inc., Hercules, CA, USA). The membranes were blocked for 1 h in Tris-Buffered Saline with Tween 20 (TBST) buffer with 5% bovine serum albumin (BSA) and were incubated with primary antibodies against GluA1 (1:1,000; Cat# 13185, RRID:AB_2732897; Cell Signaling Technology, Danvers, MA, USA) and β-actin (1:1,000; Proteintech, Rosemont, IL, USA) overnight at 4°C. Membranes were then incubated with goat anti-rabbit Ig-G secondary antibody (1:1,000; Thermo Fisher Scientific, Waltham, MA, USA) for 6 h at 4°C. An imaging system (iBright FL1000, Thermo Fisher Scientific) was used to detect the immunocomplexes by Chemiluminescence (ECL) solution (Millipore Bioscience Research Reagents, Temecula, CA, USA). The band intensity of the detected proteins was determined using the *Gels* tool in ImageJ software (version 1.50i^1^).

### Real-Time Quantitative PCR

Total mRNA was extracted from the S1BF of the cerebral cortex from WT and p53KO mice using RNAiso^TM^ Plus (9108; Takara Bio, Kusatsu, Japan) as previously reported (Feng et al., 2019). Reverse transcription (37°C for 15 min and 85°C for 5 s) from total RNA (0.5 μg) was carried out with an ABI Veriti PCR machine (Applied Biosystems, Waltham, MA, USA). cDNA was synthesized using PrimeScript^TM^ RT reagent kit (RR037A; Takara Bio) and stored at −20°C until use. qRT-PCR reactions were performed in triplicate using the SYBR^®^ Premix Ex Taq^TM^ Π kit (RR820A; Takara Bio) on a StepOnePlus thermocycler (Applied Biosystems). Expression levels of mRNA were normalized to the reference gene (β-actin) and quantified using the 2^−ΔΔCT^ method. Reactions without any template were used as negative controls. The primers used are shown in Table 1.

**Table 1:** Primers’ sequences used for qRT-PCR.

Gene	Gene bank accession	Primer	Primer sequences (5′−3′)	Product size
Grin 1	NM_008169.3	Forward	CCAGATGTCCACCAGACTAAAG	137
		Reverse	CCGTACAGATCACCTTCTTGAC	
Grin 2a	NM_008170.2	Forward	GCGCAGAACGCGGCG	261
		Reverse	AGCCTCTTGGTCCGTATCATCT	
Grin 2b	NM_008171.3	Forward	ACATGCGCTCTCCCTTAATC	259
		Reverse	AGAGATGATGGAAGTCATCTTTC	
Grin 2c	NM_010350.2	Forward	CATTGGGTCTGGCAAAGTCT	258
		Reverse	CACCTCGTTCTTCTCGTTATGG	
Grin 2d	NM_008172.2	Forward	GAGTACGACTGGACATCCTTTG	286
		Reverse	CCACCATGAACCAGACGTAG	
Grin 3a	NM_001276355.1	Forward	ATGCCAGGAAGACTGGAATATC	253
		Reverse	ATGAAGATCAGGAGGTGATAGC	
Grin 3b	NM_1340455.2	Forward	GTTTCTGAGCAACACCTCATTTC	245
		Reverse	GAGTTACCACTCGCAGCTTT	
β-actin	NM_031144	Forward	TGTCACCAACTGGGACGATA	165
		Reverse	GGGGTGTTGAAGGTCTCAAA	

### *In vitro* Cell Morphology and Dendritic Spine Density

For intracellular labeling of L5 pyramidal neurons, neurobiotin (0.1%, Vector Laboratories, Burlingame, CA, USA) was added to the intracellular solution, and the recording was maintained for at least 25 min. Subsequently, the patch pipette was slowly withdrawn. Then, slices were transferred to a container filled with 4% PFA at RT for 1 h and then at 4°C overnight for fixation. The next day, the slices were rinsed in PBS and incubated in rhodamine Red^TM^-X-conjugated streptavidin (1:1,000, Jackson ImmunoResearch Inc., West Grove, PA, USA) in PBS for 6–8 h at 4°C. After three washes in PBS, the slices were mounted and imaged with a confocal microscope (LSM 700, Zeiss, Oberkochen, Germany). Low magnification image stacks (5×, 1.5-μm *z* step) were acquired for 3D reconstruction and subsequent analysis of cell morphology. Dendritic image stacks (within the first 100 μm from the pia mater) were acquired at high magnification (63×, 0.5-μm *z* step). ZEN 2010 (Zeiss) and the *Measure* utility in ImageJ/FIJI (version 1.53c^2^) were used for the acquisition and quantification of the images, respectively.

Morphological data were obtained as previously described (Oswald et al., 2013). The cortical thickness was defined as the distance from the pia through the center of the soma to the border of L6 and white matter. Cell depth was measured from the soma center to the outermost boundary of the pia in line with the axis of the apical dendrite. To avoid variability due to small differences in slice angle and facilitate comparisons across slices, absolute cell depth (distance from pia) was normalized (cell depth/cortical depth; Yamawaki et al., 2014). Soma size was measured as the average of height and width of the cell body. Apical dendrite length was measured from the line connecting the base of the apical dendrite and the orthogonal projection to this line of the most superficial point in the tuft. The dendritic tuft height and width were measured as the rectangular dimensions defined by the point at which the apical dendrite bifurcated and the most superficial and widest points in the tuft. Morphological data were not corrected for potential tissue shrinkage effects induced by fixation. The images of dendrites from fixed tissue were analyzed to calculate the density and to classify dendritic spines according to their morphology. Spine density was calculated by dividing the total number of spines by the total length of dendrite analyzed from two to five segments per neuron. Spines were classified as mature (stubby or mushroom) or immature (thin or filopodia) according to the spine length (L), the diameter of the head (dh), and the diameter of the neck (dn) using the *Measure* utility in ImageJ/FIJI (version 1.53c^2^) software as previously described (Tyler and Pozzo-Miller, 2003; Risher et al., 2014; Liu et al., 2017). The groups were defined as follows: stubby (dn ≈ L), mushroom (dh >> dn, dh ≥ dn), thin (L >> dn), filopodia (*L* > 2 μm).

### Cranial Window Surgery

Cranial window surgery was performed as previously described (Mostany and Portera-Cailliau, 2008; Mostany et al., 2013; Voglewede et al., 2019). To prevent brain swelling and inflammation mice were injected with carprofen (5.0 mg/kg; s.c.; Zoetis Inc., Parsippany-Troy Hills, NJ, USA) and dexamethasone (0.2 mg/kg; s.c.; MWI, Boise, ID, USA) after anesthesia induction and before any incision was made. Mice were anesthetized with isoflurane (5.0% for induction, 1.5%–1.7% for maintenance), placed in a stereotaxic frame, and a 4-mm craniotomy was performed with a pneumatic dental drill over S1BF, centered at 3 mm lateral to the midline and 1.95 mm caudal to bregma. A 5-mm glass coverslip (#1; Electron Microscopy Sciences, Hatfield, PA, USA) was placed over the intact dura and secured using cyanoacrylate glue and dental acrylic (Lang Dental Mfg. Co, Inc., Wheeling, IL, USA) to the skull. A custom-made titanium bar (9.5 mm × 3.2 mm × 1.1 mm) was cemented within the dental acrylic for securing the mouse to the microscope’s stage. A 3-week recovery time was allowed before *in vivo* imaging.

### Intrinsic Optical Signal Imaging

Intrinsic optical signal (IOS) imaging was performed to precisely locate S1BF as previously described (Alexander et al., 2018; Voglewede et al., 2019). Mice were anesthetized with isoflurane (5.0% for induction, ≤1.0% for imaging) and secured to a custom-built IOS microscope using the titanium bar. IOS imaging was performed through the cranial window preparation. An image of the vasculature was taken for reference under a green (535 nm) LED array. The imaging plane was focused 250–350 μm below the dura for the imaging. IOSs from S1BF were collected using red (630 nm LEDs) illumination and a fast camera (Pantera 1M60; Dalsa, Waterloo, Canada), frame grabber (64 Xcelera-CL PX4; Dalsa), and custom-written MATLAB (RRID:SCR_001622; MathWorks, Natick, MA, USA) routines. For the stimulation, whiskers were bundled and attached with dental wax to a glass capillary tube controlled by a piezo bender actuator (Physik Instrumente, Karlsruhe, Germany). An imaging session consisted of 30 trials of whisker stimulation for 1.5 s in the rostrocaudal direction at 10 Hz with 20-s breaks. The response signal (frames 1.5 s after the onset of the stimulation) from each stimulation was divided by the baseline signal (0.9 s before stimulation) and the individual values from the 30 trials were summed to obtain the signal map. To identify the activated cortical area, an outline of the IOS map was placed over the image of the vasculature.

### Chronic *In vivo* Two-Photon Excitation Imaging

*In vivo* 2PE imaging was done as previously described (Mostany et al., 2013; Voglewede et al., 2019; Davidson et al., 2020). Mice were anesthetized with isoflurane (5.0% for induction, 1.0%–1.5% for imaging) and secured to the microscope stage. The system was a custom-built 2PE microscope using a Ti: sapphire laser (Chameleon Ultra II; Coherent, Santa Clara, CA, USA) tuned to 910 nm, a 40× 0.8 NA water-immersion objective (Olympus), and ScanImage software (RRID: SCR_014307; Pologruto et al., 2003) written in MATLAB. At the start of the chronic 2PE imaging cells were chosen from within the IOS activity map and their locations and individual dendritic fragments for a cell of interest were recorded using a coordinate system. L5 pyramidal neurons were verified by a cell body depth of 400–700 μm below the dura, and five-eight dendritic fragments of the apical tuft located in L1 were imaged chronically. High magnification image stacks (0.14 × 0.15 mm/pixel; 1.5 μm apart) were collected for the analysis of dendritic spines. Dendritic fragments were imaged chronically over imaging intervals of 4 days for a total of 5 imaging sessions. Three sessions were done before (d0, d4, d8) and two after (d12, d16) sensory-evoked synaptic plasticity induction by whisker stimulation.

### Sensory-Evoked Synaptic Plasticity Induction

To induce synaptic plasticity *in vivo* mice were subjected to rhythmic whisker stimulation as previously described (Alexander et al., 2018; Voglewede et al., 2019). Briefly, mice were anesthetized with isoflurane (5.0% for induction, <1.0% during stimulation), and their whiskers carefully bundled and attached to a piezo bender actuator, which oscillated rostrocaudally at 8 Hz continuously for 10 min. Stimulation was performed daily for four consecutive days starting on d8, and immediately after the 2PE imaging session.

### Spine Analysis

Spine density and dynamics from 2PE images were determined using spine analysis software written in MATLAB. All visible spines were annotated, including those on the *z*-axis that clearly protruded beyond the noise of the dendritic shaft. We analyzed dendritic spines from *n* = 10 cells (*N* = 8 WT mice) and *n* = 8 cells (*N* = 7 p53KO mice) and tracked a total of 5,789 and 4,393 distinct dendritic spines from WT and KO mice, respectively. For display purposes only, best projections of the dendritic segments were obtained, where the best focal plane is identified and overlaid in Adobe Photoshop CC (Adobe Systems Inc., San Jose, CA, USA), preserving all the elements in the segment, and a median filter (radius of 1) was applied. We defined the density of dendritic spines as the number of spines annotated per unit length (μm) of a dendritic segment. Spine turnover rate (TOR) was defined as the combined number of gained and lost spines per μm over a 4-day period. The fold-changes in spine density and TOR were defined as the density and TOR calculated the days after the whisker stimulation divided by the average spine density and TOR computed during the steady-state imaging regime, respectively. We defined persistent spines as those dendritic spines present at all the five imaging time points (d0 – d16). New persistent spines (NPS) were defined as newly-formed spines identified at day 4 (d4) that were still present at day 16 (d16). The survival functions of all the dendritic spines present at day 0 and of NPS were obtained by fitting their survival fraction plots to a single exponential decay curve. Survival fraction and half-life were calculated as follows: survival fraction = plateau + unstable fraction × e^−t/τ^, where *t* is the time (days) and τ (tau) is the time constant. Rate constant (*K*) = 1/τ.

### Whisker-Dependent Novel Texture Discrimination Task

We assessed whisker-dependent perceptual learning using a novel texture discrimination task following a previously described method with slight modifications (Wu et al., 2013). Mice were transported to the testing room where they were allowed to acclimate for at least 1 h. On days 1 and 2 (habituation days) mice were placed into the open arena used for the testing (a 50 cm × 50 cm × 25 cm chamber carpeted with 2 cm of standard laboratory bedding) for 10 min to habituate to the arena and promote exploration. On the 3rd day (testing day), mice were individually placed in the arena containing two identical target objects, *a* and *b* (4 cm width, 15 cm high, 0.5 cm thickness, and covered with sandpaper, of either 80 or 100 grit), and allowed to freely explore the objects for 5 min (learning phase). Then, the mice were removed and held in the transport cage for 5 min and the two objects were replaced. Replacement objects were identical in size, shape, and color and included one identical in texture to those used in the learning phase, *c*, and a second of a different grit, *d*. Mice were then placed back into the arena for the test phase, and allowed to explore for 3 min. The learning and test phases were recorded and manually analyzed with a video tracking system (Kinoscope software^3^). The amount of time mice spent actively investigating the objects was recorded and was defined as directing the nose towards the object within 2 cm. General exploratory activity was defined as the total time the mouse spent investigating the two identically textured objects during the learning phase. A discriminative activity was defined as the percentage of time spent investigating the novel texture vs. the total time spent investigating the familiar textured objects during the testing phase. Learning phase exploration index, phase in which objects *a* and *b* are identical, was calculated as (t_*b*_−t_*a*_)/(t_*a*_+t_*b*_), where t_*a*_ and t_*b*_ are the times spent exploring objects *a* and *b*, respectively. Testing phase exploration index, phase in which object *c* is identical to the objects from the learning phase, and object *d* is different from the rest of objects only in its texture, was calculated as (t_*d*_−t_*c*_)/(t_*d*_+t_*c*_), where t_*c*_ and t_*d*_ are the times spent exploring object *c* and *d*, respectively (Michaelson et al., 2018). The arena was cleaned with 70% ethanol after each test to remove olfactory cues. Behavioral analysis was performed blinded to experimental conditions.

### Statistical Analysis

Statistical analysis was performed using GraphPad Prism 8 (GraphPad Software, San Diego, CA, USA) and the Statistics Toolbox in MATLAB (version 9.4.0.813654). The Shapiro-Wilk test was used to assess the normal distribution of data. The Levene test was used to test the homogeneity of variance. The comparison of two groups was determined by paired, unpaired student’s *t*-test, or the Mann-Whitney test. The comparison of multiple groups was assessed by one-way or two-way repeated measure (RM) analysis of variance (ANOVA). Tukey *post-hoc* test was used following ANOVA test. Wilcoxon signed ranks test was used to compare the fold-change in spine density. The extra sum-of-squares F test was used to compare the best-fit values for the survival function parameters, i.e., plateau and rate constant *K*. All data are given as the mean ± SEM, unless otherwise stated. Significance was set at *p* < 0.05.

## Results

### Unaltered Gross Morphology and L5 Neuronal Density in P53^−/−^ Mice

**Figure 1:**
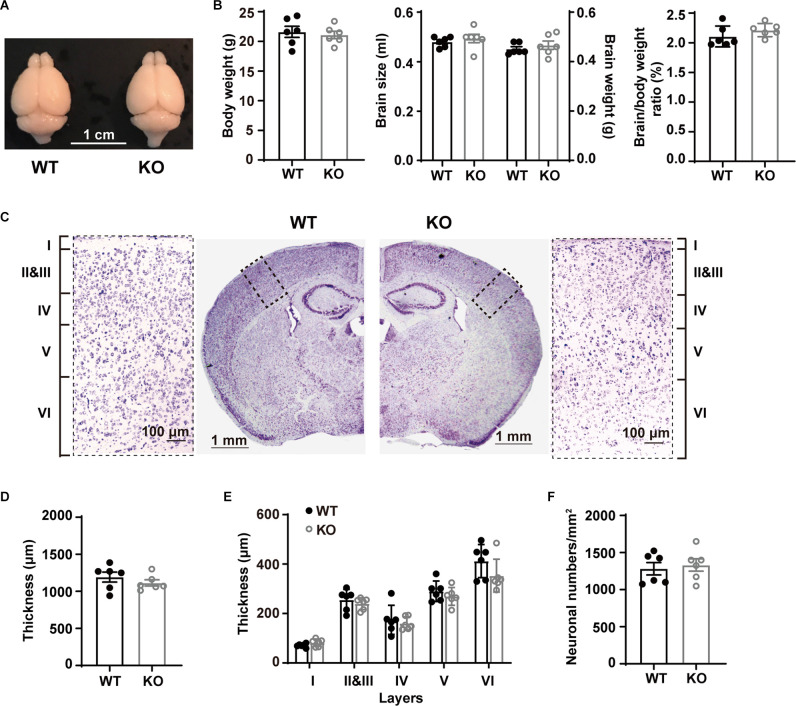
Gross morphological characterization and bodyweights of WT and p53KO mice. **(A)** Representative photographs of WT and p53KO mice brains. **(B)** Body weight, brain size, brain weight, and brain/body weight ratio of WT and KO mice. **(C)** Nissl staining of coronal brain sections and enlargement of boxed areas. Left: WT; right: KO. **(D)** Quantification of cortical thickness in S1BF. **(E)** Thickness of cortical layers I, II-III, IV, V, and VI in S1BF. **(F)** Quantification of neuron density in L5 of S1BF from mice examined in **(C)** above.

We first examined macroscopic and microscopic brain features. Whole brain comparison did not reveal apparent differences between WT and p53KO mice (Figure 1A). The body weight (*t*_(10)_ = 0.4577, *p* = 0.6570), brain size (*t*_(10)_ = 0.6984, *p* = 0.5008), brain weight (*t*_(10)_ = 0.7757, *p* = 0.4559), and the brain/body weight ratio (*t*_(10)_ = 1.275, *p* = 0.2313) of p53^−/−^ mice were comparable to those of p53^+/+^ mice (unpaired *t*-test; Figure 1B). We also compared the cortical thickness of S1BF of these mice (unpaired *t*-test; Figures 1C,D). Neither the cortical thickness (*t*_(10)_ = 0.9902, *p* = 0.3454; unpaired *t*-test; Figure 1D) nor the laminar organization (*F*_(1, 16)_ = 3.066, *p* = 0.0861; Two-way ANOVA; Figure 1E) displayed alterations between the two groups of animals. We next tested whether p53 deletion may affect L5 neuronal density in S1BF. In agreement with previous findings (Filichia et al., 2015), neuronal density in L5 did not show any difference between p53^+/+^ and p53^−/−^ mice (*t*_(10)_ = 0.4211, *p* = 0.6826, unpaired *t*-test; Figure 1F). These results suggest that p53 is not essential for the gross morphology of mouse brain.

### p53 Deficiency Alters Intrinsic Electrophysiological Properties of L5 Pyramidal Neurons in S1BF

**Figure 2:**
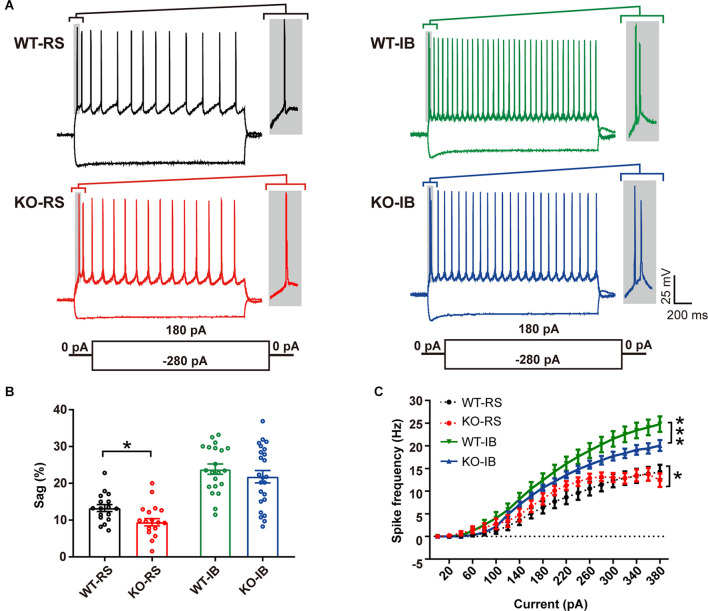
Different intrinsic electrophysiological properties of L5 pyramidal neurons of WT and KO mice. **(A)** Examples of depolarizing and hyperpolarizing traces in response to current-clamp protocols shown below traces in each cell group. In each recording the initial action potentials are given in an enlarged view on the right. **(B)** Differences in sag ratio (WT-RS vs. KO-RS, *t*_(32)_ = 2.213, *p* = 0.0342; WT-IB vs. KO-IB, *t*_(43)_ = 0.9296, *p* = 0.3577; unpaired *t*-test). **(C)** *I-f* relationships (WT-RS vs. KO-RS, *p* = 0.0202; WT-IB vs. KO-IB, *p* < 0.0001; repeated-measures two-way ANOVA, *F*_(57, 1120)_ < 0.0001). *p < 0.05; ***p < 0.001.

Despite the lack of apparent abnormalities at a gross anatomical level, it has been reported that p53KO mice suffer from several neurological problems, including learning deficiencies, anxiety, and motor deficits (Amson et al., 2000; Campana et al., 2003). Therefore, it is possible that p53 deletion may alter neuronal function without large-scale morphological changes. We tested whether p53 deficiency might affect the intrinsic properties of L5 pyramidal neurons in S1BF. L5 pyramidal neurons were categorized into regular spiking (RS) and intrinsically bursting (IB) neurons according to their firing pattern in line with previous reports (Schubert et al., 2001; Jacob et al., 2012). We recorded the responses of L5 pyramidal neurons to a series of depolarizing and hyperpolarizing current injections (Figure 2A). Membrane properties are summarized in Table 2. As indicated in prior studies, including ours (Popescu et al., 2017), and confirming the identity of the cells recorded, sag ratio, AP amplitude, and AP half-width were larger in IB neurons, whereas rheobase and AP threshold were larger in RS neurons (Supplementary Figure 1), and no significant differences were found in the V_rest_ of the two types of neurons between genotypes. Specific to the p53 deletion, the sag ratio was significantly decreased in RS neurons (Figure 2B). The *f-I* curves showed that the deletion of p53 significantly decreases the firing frequency in IB neurons. RS neurons displayed lower firing frequency rates in both genotypes (Figure 2C). These results suggest that p53 deficiency alters intrinsic electrophysiological properties of L5 pyramidal neurons in S1BF that lead to a hypoexcitable phenotype.

**Table 2:** Electrophysiological properties of layer 5 pyramidal neurons.

	WT-RS (*n* = 17)	KO-RS (*n* = 17)	WT-IB (*n* = 21)	KO-IB (*n* = 24)	Statistics
V_rest_ (mV)	−60.35 ± 1.08	−61.18 ± 1.06	−59.33 ± 1.00	−59.83 ± 0.72	
R_in_ (MΩ)	203.30 ± 25.82	209.50 ± 24.9	152.10 ± 18.04	157.90 ± 12.14	c^1^
Sag (%)	13.23 ± 0.96	10.03 ± 1.106	23.85 ± 1.39	21.80 ± 1.67	a^1^, b^3^, c^3^
Rheobase current (pA)	138.80 ± 15.29	115.30 ± 12.81	100.00 ± 9.21	86.70 ± 9.14	b^1^
AP threshold (mV)	−39.76 ± 0.79	−41.29 ± 0.89	−42.57 ± 0.89	−44.08 ± 0.71	b^1^, c^1^
AP amplitude (mV)	77.58 ± 2.27	77.73 ± 2.95	88.58 ± 1.37	86.81 ± 1.30	b^3^, c^2^
AP half-width (ms)	1.30 ± 0.04	1.46 ± 0.07	1.02 ± 0.03	1.16 ± 0.06	b^3^, c^2^

*WT-RS, Slender tuft neurons of WT mice; KO-RS, Slender tuft neurons of KO mice; WT-IB, Thick tuft neurons of WT mice; KO-IB, Thick tuft neurons of KO mice. Un-paired *t*-test on normally distributed data (mean ± SEM), otherwise non-parametric Mann-Whitney U; a, WT-RS vs. KO-RS; b, WT-RS vs. WT-IB; c, KO-RS vs. KO-IB. ^1^*p* < 0.05, ^2^*p* < 0.01; ^3^*p* < 0.001*.

### p53 Deficiency Alters Glutamatergic Synaptic Transmission in L5 Pyramidal Neurons

**Figure 3:**
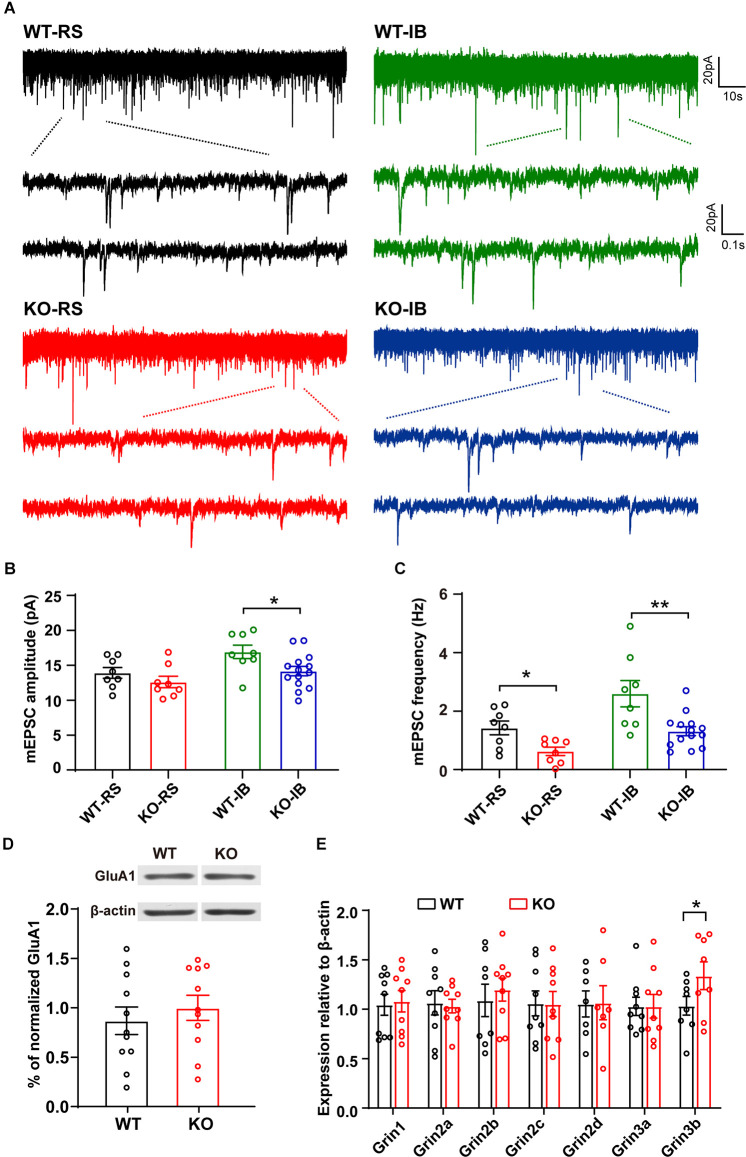
Spontaneous glutamatergic synaptic transmission in L5 pyramidal neurons of WT and KO mice. **(A)** Representative recordings of mEPSCs and two consecutive traces are shown below in an expanded time scale. **(B)** Quantification of mEPSC amplitude (WT-RS vs. KO-RS, *t*_(14)_ = 1.167, *p* = 0.2627; WT-IB vs. KO-IB, *t*_(20)_ = 2.410, *p* = 0.0257; unpaired *t*-test). **(C)** Quantification of mEPSC frequency (WT-RS vs. KO-RS, *t*_(14)_ = 2.938, *p* = 0.0108; WT-IB vs. KO-IB, *t*_(20)_ = 3.281, *p* = 0.0037; unpaired *t*-test). **(D)** Representative Western blot and the quantitative analysis of GluA1 protein (WT vs. KO, *t*_(20)_ = 0.6931, *p* = 0.4962; unpaired *t*-test). **(E)** Expression of Grin1-Grin3b mRNA (WT-Grin1 vs. KO-Grin1, *t*_(16)_ = 0.238, *p* = 0.8151; WT-Grin2a vs. KO-Grin2a, *t*_(16)_ = 0.237, *p* = 0.8160; WT-Grin2b vs. KO-Grin2b, *t*_(15)_ = 0.554, *p* = 0.5878; WT-Grin2c vs. KO-Grin2c, *t*_(16)_ = 0.040, *p* = 0.9689; WT-Grin2d vs. KO-Grin2d, *t*_(12)_ = 0.052, *p* = 0.9594; WT-Grin3a vs. KO-Grin3a, *t*_(16)_ = 0.006, *p* = 0.9951; WT-Grin3b vs. KO-Grin3b, *t*_(14)_ = 1.802, *p* = 0.0329; unpaired *t*-test). **(B,C)** Results from RS (WT, *n* = 8; KO, *n* = 8) and IB (WT, *n* = 8; KO, *n* = 14) cells from WT (*N* = 7 mice) and KO mice (*N* = 8 mice). **(D)** p53KO (*N* = 11) and WT (*N* = 11) mice. **(E)** p53KO (*N* = 7–9) and WT (*N* = 7–9) mice. *p < 0.05; **p < 0.01.

Next, to study the effect of p53 deletion on spontaneous glutamatergic synaptic transmission, we recorded mEPSCs in RS and IB neurons from p53^+/+^ and p53^−/−^ mice (Figure 3A). The amplitude and frequency of mEPSCs were generally larger in IB than in RS pyramidal neurons (Supplementary Figure 2, Table 3). The deletion of p53 significantly reduced the amplitude of mEPSCs in IB neurons (Figure 3B) and the frequency of mEPSCs in IB and RS neurons (Figure 3C). No differences were observed in the decay time of mEPSCs between p53^+/+^ and p53^−/−^ mice. These results suggest that the glutamatergic synaptic transmission is impaired in L5 pyramidal neurons when p53 is deleted.

**Table 3:** mEPSC amplitude, frequency, and decay time of layer 5 pyramidal neurons.

	WT-RS (*n* = 8)	KO-RS (*n* = 8)	WT-IB (*n* = 8)	KO-IB (*n* = 14)	Statistics
Amplitude (pA)	13.91 ± 0.77	12.60 ± 0.82	16.94 ± 0.96	14.17 ± 0.67	b^2^, c^1^
Frequency (Hz)	1.42 ± 0.23	0.63 ± 0.14	2.60 ± 0.45	1.31 ± 0.15	a^1^, b^2^, c^1^, d^2^
Decay time (ms)	2.33 ± 0.09	2.40 ± 0.22	2.53 ± 0.10	3.20 ± 0.40	

*WT-RS, Slender tuft neurons of WT mice; KO-RS, Slender tuft neurons of KO mice; WT-IB, Thick tuft neurons of WT mice; KO-IB, Thick tuft neurons of KO mice. Un-paired *t*-test on normally distributed data (mean ± SEM); a, WT-RS vs. KO-RS; b, WT-IB vs. KO-IB; c, WT-RS vs. WT-IB; d, KO-RS vs. KO-IB. ^1^*p* < 0.05, ^2^*p* < 0.01*.

The amplitude reduction of mEPSCs in p53KO mice may be due to a decreased expression of AMPAR subunit GluA1, which is a target of p53 signaling pathway (Jewett et al., 2015). We measured the expression levels of GluA1 but did not detect any significant changes in protein expression between the phenotypes (Figure 3D). Another major excitatory glutamate receptor is the NMDA receptor (NMDAR). Thus, we examined the mRNA levels of relevant NMDAR subunits and again did not find any difference in the mRNA levels of Grin1, Grin2a-2d, and Grin3a in the brains between p53^+/+^ and p53^−/−^ mice. However, we found a significant increase in Grin3b mRNA levels in p53^−/−^ brains (Figure 3E), an increase that may account for the decreased glutamatergic synaptic transmission, as previously reported (Nishi et al., 2001; Matsuda et al., 2002), of L5 pyramidal neurons in these mice (Figures 3A–C).

### p53 Is Required for the Dendritic Development of L5 Pyramidal Neurons

**Figure 4:**
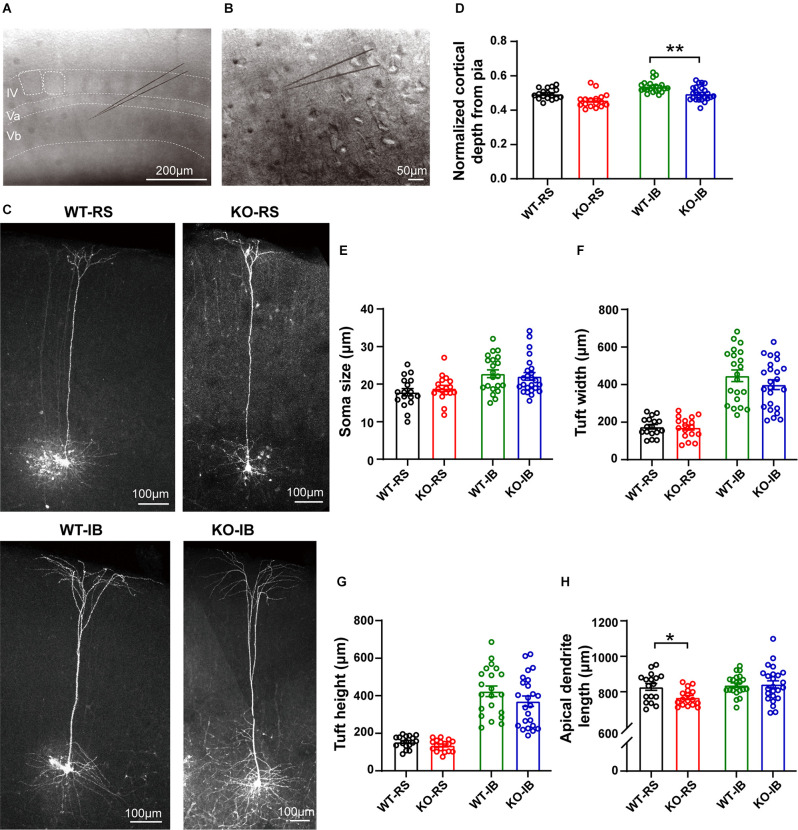
Dendritic morphology of neurobiotin-labeled L5 pyramidal neurons. **(A)** Representative image of a coronal brain section containing the barrel cortex. Note the individual barrels, either outlined and not, in layer IV. **(B)** Individual pyramidal neurons in L5 in the mouse barrel cortex were recorded using whole-cell patch-clamp technique and filled with neurobiotin. **(C)** Representative neurobiotin-labeled recorded regular spiking (RS) and intrinsically burst spiking (IB) cells from WT and KO mice. **(D)** Quantification of normalized cortical depth from pia (WT-RS vs. KO-RS, *t*_(32)_ = 1.662, *p* = 0.1063; WT-IB vs. KO-IB, *t*_(43)_ = 2.975, *p* = 0.0048; unpaired *t*-test). **(E)** Quantification of soma size (WT-RS vs. KO-RS, *t*_(32)_ = 0.7989, *p* = 0.4303; WT-IB vs. KO-IB, *t*_(43)_ = 0.4851, *p* = 0.63; unpaired *t*-test). **(F)** Quantification of tuft width (WT-RS vs. KO-RS, *t*_(32)_ = 1.712, *p* = 0.0966; WT-IB vs. KO-IB, *t*_(43)_ = 1.348, *p* = 0.1847; unpaired *t*-test). **(G)** Quantification of tuft height (WT-RS vs. KO-RS cells, *t*_(32)_ = 0.2174, *p* = 0.8293; WT-IB vs. KO-IB, *t*_(43)_ = 1.185, *p* = 0.2425; unpaired *t*-test). **(H)** Quantification of length of primary apical dendrite (WT-RS vs. KO-RS, *t*_(32)_ = 2.683, *p* = 0.0114; WT-IB vs. KO-IB, *t*_(43)_ = 0.1928, *p* = 0.848; unpaired *t*-test). Number of mice and neurons used in **(D–H)**: RS (WT, *n* = 17; KO, *n* = 17) and IB (WT, *n* = 21; KO, *n* = 24) cells from WT (*N* = 10 mice) and KO mice (*N* = 13 mice). *p < 0.05; **p < 0.01.

We then tested if p53 is required for the dendritic development of L5 pyramidal neurons. To do so, L5 pyramidal neurons were recorded and simultaneously filled with neurobiotin for *post hoc* morphological analysis and comparison between p53^+/+^ and p53^−/−^ mice (Figures 4A,B). Besides their firing pattern, L5 pyramidal neurons can also be classified into slender-tufted (ST) and thick-tufted (TT) neurons according to the morphology of their apical dendrite and dendritic tuft (Schubert et al., 2006; Hattox and Nelson, 2007; de Kock et al., 2007; Narayanan et al., 2015; Krieger et al., 2017; Popescu et al., 2017). Similar to our previous study (Popescu et al., 2017), RS and IB neurons corresponded to the ST and TT neurons, respectively. As shown in Figure 4C, RS (ST) neurons had a less complex and shallow apical tuft than IB (TT) neurons, regardless of the genotype.

In order to compare the layer profile of RS and IB neurons, we normalized the depth from the pia of each soma to the cortical thickness. S1BF L5 RS neurons occurred at a normalized depth of 0.55 ± 0.01 and 0.53 ± 0.01, n.s., in WT and p53KO mice, respectively. However, IB neurons in KO mice appeared to be more superficially located than those from WT mice (0.59 ± 0.01 vs. 0.55 ± 0.01, WT vs. KO, respectively; *t*_(43)_ = 2.975, *p* = 0.0048; unpaired *t*-test; Figure 4D, Table 4). Morphologically, IB neurons were distinguished from RS neurons by their larger soma size and longer and wider dendritic tuft (Supplementary Figure 3). However, for a given neuron subtype, there were no differences between the two genotype groups (Figures 4E–G). Apical dendrite length was significantly shorter in KO-RS neurons than that in WT-RS neurons (Figure 4H).

**Table 4:** Morphological characteristics of layer V pyramidal neurons.

	WT-RS (*n* = 17)	KO-RS (*n* = 17)	WT-IB (*n* = 21)	KO-IB (*n* = 24)	Statistics
Cell depth^∫^	0.55 ± 0.01	0.53 ± 0.01	0.59 ± 0.01	0.55 ± 0.01	b^2^, c^2^
Soma size (μm)	17.85 ± 0.97	18.87 ± 0.83	22.76 ± 1.05	22.06 ± 1.00	c^2^, d^1^
Apical dendrite length (μm)	827.50 ± 19.10	767.70 ± 11.47	837.40 ± 12.82	842.10 ± 20.17	a^1^, d^2^
Tuft height (μm)	153.60 ± 7.74	135.10 ± 7.56	424.00 ± 28.26	370.90 ± 27.36	c^3^, d^3^
Tuft width (μm)	175.00 ± 12.08	171.00 ± 13.62	447.10 ± 30.80	399.70 ± 25.96	c^3^, d^3^

*WT-RS, Slender tuft neurons of WT mice; KO-RS, Slender tuft neurons of KO mice; WT-IB, Thick tuft neurons of WT mice; KO-IB, Thick tuft neurons of KO mice. Un-paired *t*-test on normally distributed data (mean ± SEM); a, WT-RS vs. KO-RS; b, WT-IB vs. KO-IB; c, WT-RS vs. WT-IB; d, KO-RS vs. KO-IB. ^1^*p* < 0.05, ^2^*p* < 0.01; ^3^*p* < 0.001. ^∫^, Normalized to cell depth from the pia*.

**Figure 5:**
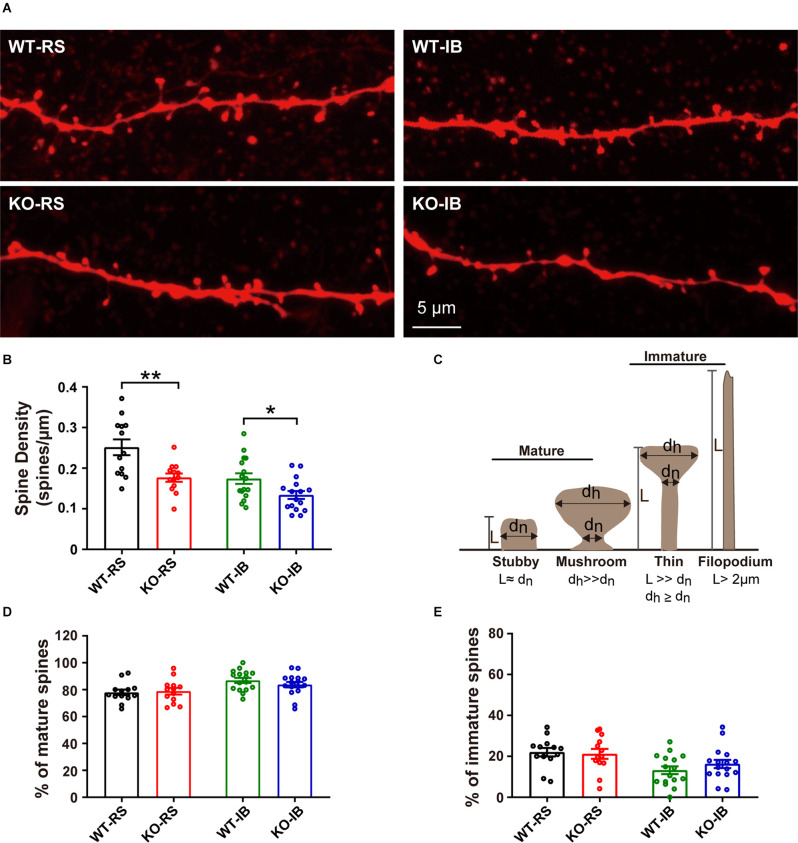
Density of dendritic spines of L5 pyramidal neurons in S1BF of adult WT and KO mice. **(A)** Representative images of dendrites in RS and IB neurons from p53KO and WT mice. **(B)** Quantification of spine density (WT-RS vs. KO-RS, *t*_(24)_ = 3.396, *p* = 0.0024; WT-IB vs. KO-IB, *t*_(30)_ = 2.444, *p* = 0.0206; unpaired *t*-test). **(C)** Schematic representation of morphological classification of dendritic spines. **(D)** Quantification of percentages of mature spine (WT-RS vs. KO-IB, *t*_(24)_ = 0.2571, *p* = 0.7993; WT-IB vs. KO-IB, *t*_(30)_ = 1.107, *p* = 0.2772; WT-RS vs. WT-IB, *t*_(27)_ = 3.142, *p* = 0.0040; KO-RS vs. KO-IB, *t*_(27)_ = 1.552, *p* = 0.1324; unpaired *t*-test). **(E)** Quantification of percentages of immature spine (WT-RS vs. KO-RS, *t*_(24)_ = 0.2571, *p* = 0.7993; WT-IB vs. KO-IB, *t*_(30)_ = 1.107, *p* = 0.2772; WT-RS vs. WT-IB, *t*_(27)_ = 3.142, *p* = 0.0040; KO-RS vs. KO-IB, *t*_(27)_ = 1.552, *p* = 0.1324; unpaired *t*-test). Number of mice and neurons used in **(B,D,E)**: from RS (WT, *n* = 13; KO, *n* = 13) and IB (WT, *n* = 16; KO, *n* = 16) cells in WT (*N* = 6) and KO mice (*N* = 9). *p < 0.05; **p < 0.01.

Given that dendritic spines are the main input of excitatory transmission in pyramidal neurons, we postulated that the decrease in mEPSC frequency in KO mice may result from a reduction in spine density. To test this speculation, we compared the dendritic spine density of the neurons recorded from p53^+/+^ and p53^−/−^ mice (Figure 5A). Spine density in p53KO mice was significantly decreased in both RS and IB neurons compared to those of WT mice (WT-RS, 0.25 ± 0.02 vs. KO-RS, 0.18 ± 0.01; WT-IB, 0.17 ± 0.01 vs. KO-IB, 0.13 ± 0.01; Figure 5B). We next classified the spines into mature (stubby or mushroom) or immature (thin or filopodia) spines (Figure 5C) as previously described (Fiala et al., 1998; Risher et al., 2014). The percentages of mature and immature spines were not significantly different between genotypes for either spine category (Figures 5D,E). Together, these data suggest that p53 may be required for the dendritic development of L5 pyramidal neurons of S1BF and involved in spinogenesis. The latter set up the motivation for the *in vivo* imaging studies, aimed to study the dynamics of dendritic spines.

### Steady-State and Sensory-Evoked Dynamics of Dendritic Spines Are Altered in p53KO Mice

**Figure 6:**
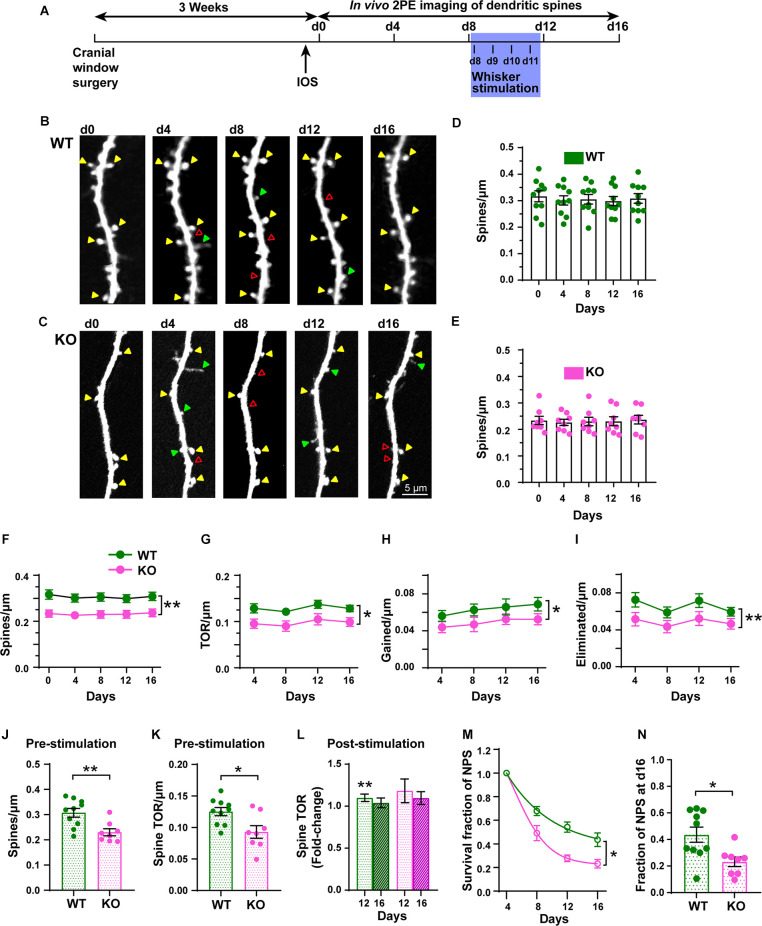
Chronic *in vivo* quantification of density and dynamics of dendritic spines of L5 pyramidal neurons in S1BF. **(A)** Experimental timeline with imaging intervals of 4 days over 16 days (d0 to d16). Whisker stimulation occurred daily from d8 (following 2P imaging) until d11. IOS: Intrinsic Optical Signal imaging. **(B,C)** Representative high-resolution images of dendritic segments from the apical tuft of layer 5 pyramidal neurons in WT and KO mice acquired using *in vivo* 2PE microscopy. **(D,E)** Density of dendritic spines in WT and KO mice. **(F–I)** Changes in spine density, TOR/μm, gained/μm, and eliminated/μm at 4-day intervals in WT and KO mice. **(J,K)** Pre-stimulation dendritic spine density and TOR ratios. **(L)** Post-stimulation (pre-stim. vs. d12 and d16) fold-change of TOR. **(M)** Survival function of new persistent spines (NPS) formed at d4. **(N)** Fraction of NPS at d16. Arrowheads indicate persistent (yellow), gained (green), and eliminated (red) spines. *p < 0.05; **p < 0.01.

To examine dendritic spine dynamics of L5 pyramidal neurons from both WT and p53KO mice during steady-state and after plasticity-inducing conditions, we used chronic *in vivo* 2PE microscopy through a cranial window (Figure 6A). We imaged the same dendritic segments of individually identified L5 pyramidal neurons for three baseline sessions (d0, d4, d8; steady-state) before the introduction of daily whisker stimulations (d8-d11) and after whisker stimulation (d12 and d16; stimulated state). Time-lapse imaging every 4 days over a total of 16 days showed the dynamic nature (gains and losses) of dendritic spines in both WT and KO mice, including persistent, newly formed, and eliminated spines (Figures 6B,C). The spine density was not significantly different across the duration of the experiment in either WT or KO mice (Figures 6D–F). However, the density of dendritic spines (*F*_(1, 16)_ = 10.65, *p* = 0.0049, RM two-way ANOVA), TOR/μm (*F*_(1, 16)_ = 8.406, *p* = 0.0105, RM two-way ANOVA), number of gained spines/μm (*F*_(1, 16)_ = 5.899, *p* = 0.0273, RM two-way ANOVA), and number of eliminated spines/μm (*F*_(1, 16)_ = 9.416, *p* = 0.0073, RM two-way ANOVA) were significantly decreased in the p53KO mice compared to WT mice (Figures 6F–I). Subsequent analysis at different time points found a significant lower spine density in KO compared with WT mice (*p* = 0.0064 for d0, *p* = 0.0177 for d4, *p* = 0.0154 for d8, *p* = 0.0364 for d12, *p* = 0.0246 for d16; Sidak’s multiple comparisons test; not shown), whereas no significant differences after *post hoc* analysis were computed for TOR/μm, gained spines/μm, and eliminated spines/μm at the different time points.

To determine the role of p53 in structural plasticity, we used a 4-day interval and sampled the same dendritic fragments pre- and post-stimulation. Data from the steady-state imaging sessions were averaged to serve as a baseline (Pre) for comparison with the data obtained the days after the whisker stimulation (d12 and d16). KO mice displayed lower pre-stimulation spine density than WT mice did (Figure 6J, *t*_(16)_ = 3.365, *p* = 0.0039; unpaired *t*-test). The TOR of dendritic spines was also significantly decreased in KO mice compared with WT mice (Figure 6K, *t*_(16)_ = 2.809, *p* = 0.0126; unpaired *t*-test). While the number of gained spines per μm was not significantly different between genotypes (Supplementary Figure 4A; *t*_(16)_ = 1.893, *p* = 0.0765; unpaired *t*-test), the linear density of eliminated spines was reduced in p53KO mice compared with WT mice (Supplementary Figure 4B; *t*_(16)_ = 2.437, *p* = 0.0286; unpaired *t*-test). Whisker stimulation did not affect the density of dendritic spines (Supplementary Figure 4D). However, while the changes in TOR after the stimulation relative to the steady-state were significantly increased at d12 in WT mice (WT-pre vs. d12, *t*_(9)_ = 3.737, *p* = 0.005; paired *t*-test; Figure 6L) and returned to normal levels at d16, similar to previous results (Voglewede et al., 2019), we could not detect significant changes in TOR compared to baseline in p53KO mice (p53KO-pre vs. d12, *t*_(7)_ = 1.372, *p* = 0.2124; paired *t*-test; Supplementary Figure 4D). We did not detect statistically significant differences in the fold-change of gained and eliminated spines (Supplementary Figures 4D,E). Finally, we analyzed the survival functions of the dendritic spines identified on the first day of imaging and of new persistent spines (NPS) identified on d4. When all the spines scored at d0 were followed over time we did not find differences in the rate constant (*K*) of the exponential decay fits computed for both groups (WT, 0.042 ± 0.002; KO, 0.038 ± 0.002; *F*_(1, 88)_ = 1.996; *p* = 0.7179; mean ± SD; extra sum-of-squares F test; Supplementary Figure 4F). These *K* values translated into half-life values of 16.6 ± 0.6 and 18.3 ± 1.1 days (mean ± SD) for dendritic spines of WT and p53KO mice, respectively, and the fraction of persistent spines at day 16 for both groups was not different either (WT, 0.57 ± 0.02; KO, 0.60 ± 0.03; *t*_(16)_ = 1.009, *p* = 0.3280; extra sum-of-squares F test; Supplementary Figure 4G). Regarding NPS, *K* values were significantly different (WT: 0.076 ± 0.006; KO: 0.154 ± 0.011; *F*_(1, 70)_ = 44.57; *p* < 0.0001; mean ± SD; extra sum-of-squares F test; Figure 6M), representing NPS half-life values of 9.1 ± 0.7 and 4.5 ± 0.3 days (mean ± SD) for WT and KO mice, respectively. In fact, the fraction of d4 NPS still present the last day of imaging was significantly reduced in p53KO mice (0.23 ± 0.04) compared to WT mice (0.44 ± 0.06; *t*_(16)_ = 2.839, *p* = 0.0118; unpaired *t*-test; Figure 6N). These results indicate that p53 deficiency leads to the alteration of both steady-state and sensory-evoked dynamics of dendritic spines in L5 pyramidal neurons of S1BF.

### Texture Discrimination Ability Is Impaired in p53^−/−^ Mice

**Figure 7:**
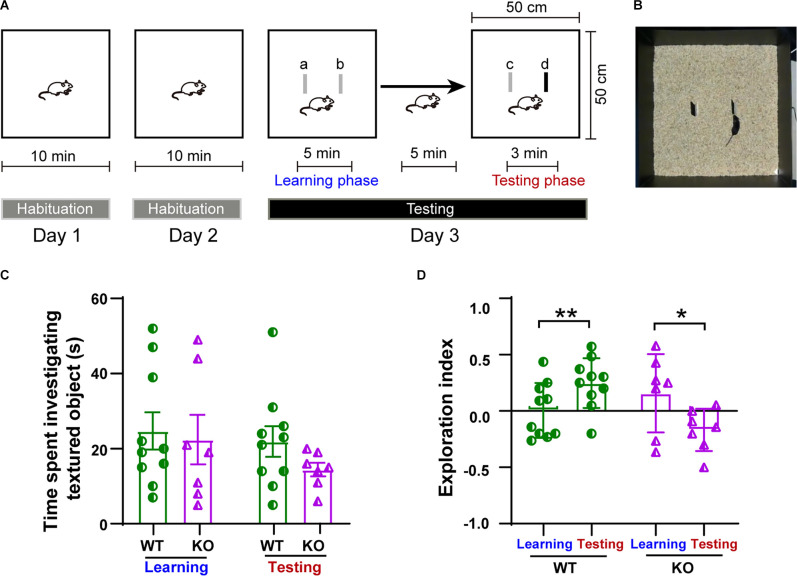
Impairment in whisker-dependent novel texture discrimination task in p53KO mice. **(A)** Schematic of the protocol for the texture discrimination task. **(B)** Photograph of the testing apparatus. **(C)** Total time mice spent investigating the texture (*F*_(3, 30)_ = 0.8077, *p* = 0.4996, one-way ANOVA followed by *post hoc* Tukey’s test). **(D)** Scatter plot showing exploration index for animals in **(C)**: WT (paired *t*-test: *t*_(9)_ = 4.206, *p* = 0.0023; one-sample *t*-test: WT learning, *t*_(9)_ = 0.0602, *p* = 0.9533; Wt-testing, *t*_(9)_ = 3.561, *p* = 0.0061). KO (paired *t*-test: *t*_(6)_ = 3.646, *p* = 0.0108; one-sample *t*-test: KO learning, *t*_(6)_ = 1.197, *p* = 0.2763; KO testing, *t*_(6)_ = 2.374, *p* = 0.0552). *p < 0.05; **p < 0.01.

To assess whether the impairment in plasticity observed in the p53KO group could lead to the impairment of whisker-mediated contextual learning, we performed a whisker-dependent novel texture discrimination task on p53^+/+^ and p53^−/−^ mice (Figures 7A,B). During either the learning or the testing phase on day 3, the amount of time spent investigating the textured objects was not significantly different between two groups of mice (*F*_(3, 30)_ = 0.8077, *p* = 0.4996, one-way ANOVA followed by *post hoc* Tukey’s test; Figure 7C). During testing with the novel textured object, p53^+/+^ mice were able to discriminate and spent significantly more time exploring the new textured object (paired *t*-test: *t*_(9)_ = 4.206, *p* = 0.0023; one-sample *t*-test: WT learning, *t*_(9)_ = 0.0602, *p* = 0.9533; WT testing, *t*_(9)_ = 3.561, *p* = 0.0061, Figure 7D). In contrast, P53^−/−^ mice had a harder time discriminating between the novel and the familiar texture during the testing phase (paired *t*-test: *t*_(6)_ = 3.646, *p* = 0.0108; one-sample *t*-test: KO learning, *t*_(6)_ = 1.197, *p* = 0.2763; KO testing, *t*_(6)_ = 2.374, *p* = 0.0552, Figure 7D). These data suggest that the deletion of p53 impairs whisker-dependent texture discrimination and contextual learning.

## Discussion

In this study, we identified unknown roles of p53 in structural, functional, and synaptic properties of L5 pyramidal neurons in S1BF. Our findings indicate that deficiency of p53 leads to the suppression of excitability of L5 pyramidal neurons, decrease of apical spine density and turnover ratio, and impairment of performance in a whisker-dependent task.

The tumor suppressor p53 is activated following stress and has been intensively studied in human diseases (Hu et al., 2007; Checler and Alves da Costa, 2014; Kastenhuber and Lowe, 2017). Besides displaying spontaneous tumors by 6 months of age (Donehower et al., 1992; Jacks et al., 1994), a fraction of p53KO mice presents detectable defects in embryonic neuron development and exhibit exencephaly due to failure of neural tube closure (Armstrong et al., 1995; Sah et al., 1995). These mice are not able to survive beyond birth. The rest of the p53KO mice are generally viable and healthy, with normal body weight (Campana et al., 2003; Molchadsky et al., 2013). As expected, we did not find significant differences in body weight, brain size, brain weight, the brain/body weight ratio, cortical thickness, and the thickness of cortical layers between WT and p53KO young adult mice. It is well known that p53 can induce apoptosis once activated (Fridman and Lowe, 2003), suggesting that neuronal numbers may be higher in p53KO mice. In this regard, a reduction of the cortex thickness of more than 50% in a fraction of p53KO mice compared to WT mice has been described (Amson et al., 2000), while another report has shown that the number of Purkinje, granule, and inferior olivary cells of p53KO mice were similar to that of control mice (Campana et al., 2003). Consistently with this latter study, we did not observe differences in neuron density in L5 of S1BF between the two genotypes.

L5 pyramidal neurons are the main integrators of information in the cortical column, spanning all the layers of the cortex and projecting to other cortical areas as well as to subcortical structures. These neurons are usually classified by their layer location (L5a and L5b), projection (corticothalamic, corticotrigeminal, corticostriatal, and callosal), morphology (ST and TT), and firing pattern (RS and IB; Manns et al., 2004; Hattox and Nelson, 2007; Oberlaender et al., 2012; Narayanan et al., 2015, 2017; Krieger et al., 2017). In line with previous reports, we found a close association between firing pattern and cell morphology. IB cells had larger somas and thicker dendritic tufts (TT), and predominantly occupied the lower portion of L5 (L5b). In contrast, RS cells were characterized by a smaller soma, a slender dendritic tuft (ST), and preferentially occupied a more superficial layer location (L5a).

Hyperpolarization-activated cyclic nucleotide-gated (HCN) channels are voltage and ligand (cAMP)-activated membrane channels that contribute to neuronal electrical excitability (Biel et al., 2009), and HCN channel-induced sag is one of the main intrinsic parameters of L5 pyramidal neurons in S1BF (Popescu et al., 2017). Our results revealed a reduction in the sag ratio (RS cells) and spike frequency (IB cells) in p53KO mice compared to WT mice, suggesting a decrease in excitability and an overall decrease in excitatory outputs. Although the underlying mechanism remains to be elucidated, we speculate on a possible molecular mechanism involving neural precursor cells expressed developmentally down-regulated gene 4-like (Nedd4-2 or NEDD4L). Nedd4-2 is a ubiquitin ligase that targets HCN and sodium channels for ubiquitination (Manning and Kumar, 2018), and *in vivo* and *in vitro* studies have shown that Nedd4-2 binds to and down-regulates surface expression of HCN1 channels (Wilkars et al., 2014). Accordingly, HCN current is reduced when HCN1 and Nedd4-2 are co-expressed in Xenopus oocytes (Wilkars et al., 2014). Nedd4-2 is also a target gene of p53, and its expression is elevated when p53 is inhibited (Wei et al., 2006; Jewett et al., 2015, 2016). Thus, the expression of Nedd4-2 may be increased in p53KO mice, leading to decreased sag ratio and firing frequencies. Further studies are needed to test this possibility. We also found that the frequency of mEPSCs is reduced in KO-RS and KO-IB L5 neurons, suggesting a decrease in functional excitatory inputs. These results indicate that the activity of the sensory processing pathway is weakened in p53KO mice. The results regarding the function of glutamatergic synapses suggest that p53 may play a role in regulating glutamatergic synapse development. GluA1 (a subunit of the AMPA receptor) and NMDA receptors have been demonstrated to be involved in p53-related synaptic plasticity (Hori et al., 2005; Lee et al., 2018). However, we did not find any alteration of the protein levels of GluA1 and the mRNA levels of Grin1, Grin2a-2d, and Grin3a between genotypes. Instead, we found a significant increase in Grin3b mRNA levels, which encodes for the GluN3B subunit of the NMDA receptor (i.e., NR3B). The fact that glutamate-evoked currents are decreased in cells co-expressing the NR3B together with the NR1 and NR2A, compared to cells expressing NR1 and NR2A without NR3B (Matsuda et al., 2002) suggests that the Grin3b mRNA up-regulation observed in p53KO mice may account for the decreased activity of L5 pyramidal neurons observed in our functional studies.

Activation of p53 has been shown to increase spinogenesis in rat hippocampal neuronal cultures (Choi et al., 2014). In contrast, the upregulation of MDM2 proto-oncogene, a p53 inhibitor, reduces the density of dendritic spines of granule cells in the olfactory bulb (Yoshihara et al., 2014). In accord with a previous study (Oberlaender et al., 2012), we found that the spine density of RS neurons is higher than that of IB neurons, regardless of the genotype, and that the density is lower in both KO-RS and KO-IB neurons than in WT littermates. These results are well in line with the findings from the mEPSC analysis indicating a reduction in the frequency of mEPSCs. The analysis of the shape of the dendritic protrusions, in which we did not observe changes in the proportions of mature or immature spines between phenotypes, suggests that p53 may not be critical for the maturation of dendritic spines. Altogether, our results indicate that while p53 may not be involved in spine maturation, it may be playing a critical role in spinogenesis and structural synaptic plasticity. However, and based on the data obtained throughout the study, we cannot suggest that one subtype of L5 pyramidal neuron is more affected than the other by the deletion of the gene.

The results from the *in vitro* studies, indicating a reduction in dendritic spines, set up the motivation for *in vivo* studies aiming to investigate dynamic events of these synaptic structures during baseline conditions and after a sensory manipulation intended to increase spine turnover. Our *in vivo* approach confirmed the reduced density of dendritic spines in projection L5 neurons of p53KO mice observed in the histological analyses. We could also determine that, similar to our previous studies in wildtype mice (Alexander et al., 2018; Voglewede et al., 2019), whisker stimulation does not alter spine density in either genotype. Regarding dendritic spine dynamics, we examined how the deficiency in p53 affects spine turnover and whether p53 could play an important role in experience-dependent structural plasticity in sensory circuits. In baseline conditions, L5 pyramidal neurons from p53KO mice presented a lower rate of spine turnover, due to a decrease in the rates of both gained and eliminated spines. This stagnancy of the remodeling of dendritic spines may be a consequence of the deficient excitatory drive of these neurons detected in our electrophysiology assessments. Also, based on our previous studies in aged mice where the inhibitory function is declined and the baseline TOR of dendritic spines is increased (Mostany et al., 2013; Voglewede et al., 2019; Popescu et al., 2021), we speculate that an increase in inhibitory drive may be altering the normal dynamics of dendritic spines in p53KO mice. After whisker stimulation, the TOR of spines increased in WT mice, as we have shown previously (Alexander et al., 2018; Voglewede et al., 2019). However, this effect was blunted in p53KO mice, suggesting again a resistance to synaptic remodeling, and therefore limiting the ability of the cortical circuits to incorporate new information. This lack of plasticity, or the mechanism underlying the limited plasticity, may be responsible for the deficits in the whisker-dependent contextual learning task detected in the study. Additionally, our results indicate that the persistence of newly-formed dendritic spines is reduced in the p53KO mice, suggesting an important role of p53 in synaptic stabilization and further hinting at adeficiency to store/recall information of the cortical circuits of the mutant mice. Overall, the results obtained in the present study suggest an important role for p53 in the regulation of synaptic plasticity, as indicated by the reduced excitability and density of dendritic spines observed in L5 pyramidal neurons of p53KO mice. Nevertheless, further studies utilizing more specific manipulations of the expression of p53, as well as experiments designed to decipher the potential role of the p53-mediated Nedd4 expression regulation, which seems to regulate HCN channels, and therefore neuronal excitability, are needed to determine the specific mechanism or mechanisms by which p53 regulates structural and functional plasticity of pyramidal neurons, and maybe of other neuron subtypes.

Although not being pursued in this study, the results from p53KO mice are in many aspects opposed to the results that our laboratory has observed over the recent years from L5 pyramidal neurons in aged mice, i.e., hyperexcitability (Popescu et al., 2021), and increased density and TOR of dendritic spines (Mostany et al., 2013; Voglewede et al., 2019). Together with reports indicating an increased expression of p53 in aged human cells (Kulju and Lehman, 1995; Bitto et al., 2010), our data may suggest an increased expression of this transcription factor in aged neurons.

In summary, this study is the first to examine the effect of p53 on the function and spine formation both *in vitro* and *in vivo* in L5 pyramidal neurons of the somatosensory cortex. By regulating both the anatomical and physiological features of L5 pyramidal neurons, p53 appears to play a crucial role in S1BF-dependent behaviors.

## Data Availability Statement

The raw data supporting the conclusions of this article will be made available by the authors, without undue reservation.

## Ethics Statement

The animal study was reviewed and approved by the Institutional Animal Care and Use Committees at Tulane University School of Medicine and Nanchang University.

## Author Contributions

TL, HL, and RM designed the research. HK, TL, CJ, JWa, SW, SZ, AD, and RM performed the research. HK, TL, JWu, SP, HL, and RM analyzed the data. HK, TL, AD, HL, and RM wrote the manuscript. All authors contributed to the article and approved the submitted version.

## Conflict of Interest

The authors declare that the research was conducted in the absence of any commercial or financial relationships that could be construed as a potential conflict of interest.

## Publisher’s Note

All claims expressed in this article are solely those of the authors and do not necessarily represent those of their affiliated organizations, or those of the publisher, the editors and the reviewers. Any product that may be evaluated in this article, or claim that may be made by its manufacturer, is not guaranteed or endorsed by the publisher.

## Footnotes

^1^ http://rsb.info.nih.gov/ij/

^2^ https://fiji.sc

^3^ https://sourceforge.net/projects/kinoscope/

## Supplementary Material

Supplementary Figure 1 Intrinsic electrophysiological properties of L5 pyramidal neurons of WT and KO mice. **(A)** Sag ratio (WT-RS vs. WT-IB, *t*_(36)_ = 5.985, *p* < 0.0001; KO-RS vs. KO-IB, *t*_(39)_ = 5.356, *p* < 0.0001; unpaired *t*-test). **(B)** Rheobase current (WT-RS vs. KO-RS, *t*_(32)_ = 1.180, *p* = 0.2468; WT-IB vs. KO-IB, *t*_(43)_ = 1.023, *p* = 0.3118; WT-RS vs. WT-IB, *t*_(36)_ = 2.267, *p* = 0.0295; KO-RS vs. KO-IB, *t*_(39)_ = 1.872, *p* = 0.0687; unpaired *t*-test). **(C)** AP threshold (WT-RS vs. KO-RS, *t*_(32)_ = 1.290, *p* = 0.2064; WT-IB vs. KO-IB, *t*_(43)_ = 1.349, *p* = 0.1844; WT-RS vs. WT-IB, *t*_(36)_ = 2.317, *p* = 0.0263; KO-RS vs. KO-IB, *t*_(39)_ = 2.479, *p* = 0.0176; unpaired *t*-test). **(D)** AP amplitude (WT-RS vs. KO-RS, *t*_(32)_ = 0.0370, *p* = 0.9707; WT-IB vs. KO-IB, *t*_(43)_ = 0.9384, *p* = 0.3533; WT-RS vs. WT-IB, *t*_(36)_ = 3.856, *p* = 0.0005; KO-RS vs. KO-IB, *t*_(39)_ = 3.119, *p* = 0.0034; unpaired *t*-test). **(E)** AP half-width (WT-RS vs. KO-RS, *p* = 0.0932; WT-IB vs. KO-IB, *p* = 0.1077; WT-RS vs. WT-IB, *p* < 0.0001; KO-RS vs. KO-IB, *p* < 0.0018; Mann-Whitney test). **(F)** *I-f* relationships (WT-RS vs. WT-IB, *p* < 0.0001; KO-RS vs. KO-IB, *p* < 0.0001; repeated-measures Two-way ANOVA, *F*_(57,1120)_ < 0.0001). **p* < 0.05; ***p* < 0.01; ****p* < 0.001.

Supplementary Figure 2 Spontaneous glutamatergic synaptic transmission in L5 pyramidal neurons of WT and KO mice. **(A)** Quantification of mEPSC amplitude from RS (WT, *n* = 8; KO, *n* = 8) and IB (WT, *n* = 8; KO, *n* = 14) cells from WT (*N* = 7 mice) and KO mice (*N* = 8 mice; WT-RS vs. WT-IB, *t*_(14)_ = 2.454, *p* = 0.0278; KO-RS vs. KO-IB, *t*_(20)_ = 1.444, *p* = 0.1642; unpaired *t*-test). **(B)** Quantification of mEPSC frequency (WT-RS vs. WT-IB, *t*_(14)_ = 2.313, *p* = 0.0364; KO-RS vs. KO-IB, *t*_(20)_ = 2.973, *p* = 0.0075; unpaired *t*-test). **p* < 0.05; ***p* < 0.01.

Supplementary Figure 3 Dendritic morphology of neurobiotin-labeled L5 pyramidal neurons. **(A)** Quantification of normalized cortical depth from pia (WT-RS vs. WT-IB, *t*_(36)_ = 2.809, *p* = 0.008; KO-RS vs. KO-IB, *t*_(39)_ = 1.659, *p* = 0.1051; unpaired *t*-test). **(B)** Quantification of soma size (WT-RS vs. WT-IB, *t*_(36)_ = 3.368, *p* = 0.0018; KO-RS vs. KO-IB, *t*_(39)_ = 2.315, *p* = 0.0260; unpaired *t*-test). **(C)** Quantification of tuft width (WT-RS vs. WT-IB, *t*_(36)_ = 8.386, *p* < 0.0001; KO-RS vs. KO-IB, *t*_(39)_ = 7.093, *p* < 0.0001; unpaired *t*-test). **(D)** Quantification of tuft height (WT-RS vs. WT-IB, *t*_(36)_ = 7.560, *p* < 0.0001; KO-RS vs. KO-IB, *t*_(39)_ = 6.932, *p* < 0.0001; unpaired *t*-test). **(E)** Quantification of length of primary apical dendrites (WT-RS vs. WT-IB, *t*_(36)_ = 0.4417, *p* = 0.6614; KO-RS vs. KO-IB, *t*_(39)_ = 2.872, *p* = 0.0066; unpaired *t*-test). **(F)** Quantification of spine density (WT-RS vs. WT-IB, *t*_(27)_ = 3.394, *p* = 0.0021; KO-RS vs. KO-IB, *t*_(27)_ = 2.977, *p* = 0.0061;unpaired *t*-test). **(G)** Quantification of percentages of mature spine (WT-RS vs. KO-IB, *t*_(24)_ = 0.2571, *p* = 0.7993; WT-IB vs. KO-IB, *t*_(30)_ = 1.107, *p* = 0.2772; WT-RS vs. WT-IB, *t*_(27)_ = 3.142, *p* = 0.0040; KO-RS vs. KO-IB, *t*_(27)_ = 1.552, *p* = 0.1324; unpaired *t*-test). **(H)** Quantification of percentages of immature spine (WT-RS vs. KO-RS, *t*_(24)_ = 0.2571, *p* = 0.7993; WT-IB vs. KO-IB, *t*_(30)_ = 1.107, *p* = 0.2772; WT-RS vs. WT-IB, *t*_(27)_ = 3.142, *p* = 0.0040; KO-RS vs. KO-IB, *t*_(27)_ = 1.552, *p* = 0.1324; unpaired *t*-test). The number of mice and neurons used in **(A–E)** of RS (WT, *n* = 17; KO, *n* = 17) and IB (WT, *n* = 21; KO, *n* = 24) cells from WT (*N* = 10 mice) and KO mice (*N* = 13 mice). **(F–H)** RS (WT, *n* = 13; KO, *n* = 13) and IB (WT, *n* = 16; KO, *n* = 16) cells in WT (*N* = 6) and KO mice (*N* = 9). **p* < 0.05; ***p* < 0.01; ****p* < 0.001.

Supplementary Figure 4 Chronic *in vivo* quantification of density and dynamics of dendritic spines of L5 pyramidal neurons in S1BF of WT and p53KO mice before and after stimulation. **(A,B)** Pre-stimulation dendritic spine gained and eliminated ratios. **(C–E)** Post-stimulation (pre-stim. vs. d12 and d16) fold-change of dendritic spine density **(C)**, gained spines **(D)**, and eliminated spines **(E)**. **(F)** Survival function of all the spines present at d0. **(G)** Fraction of persistent spines at d16. **p* < 0.05.
