# Supplementary figures and images for: Genetic Deficiency of p53 Leads to Structural, Functional, and Synaptic Deficits in Primary Somatosensory Cortical Neurons of Adult Mice
Source: Front Mol Neurosci. 2022 Apr 7;15:871974. doi: 10.3389/fnmol.2022.871974 (PMC9021533; doi:10.3389/fnmol.2022.871974)

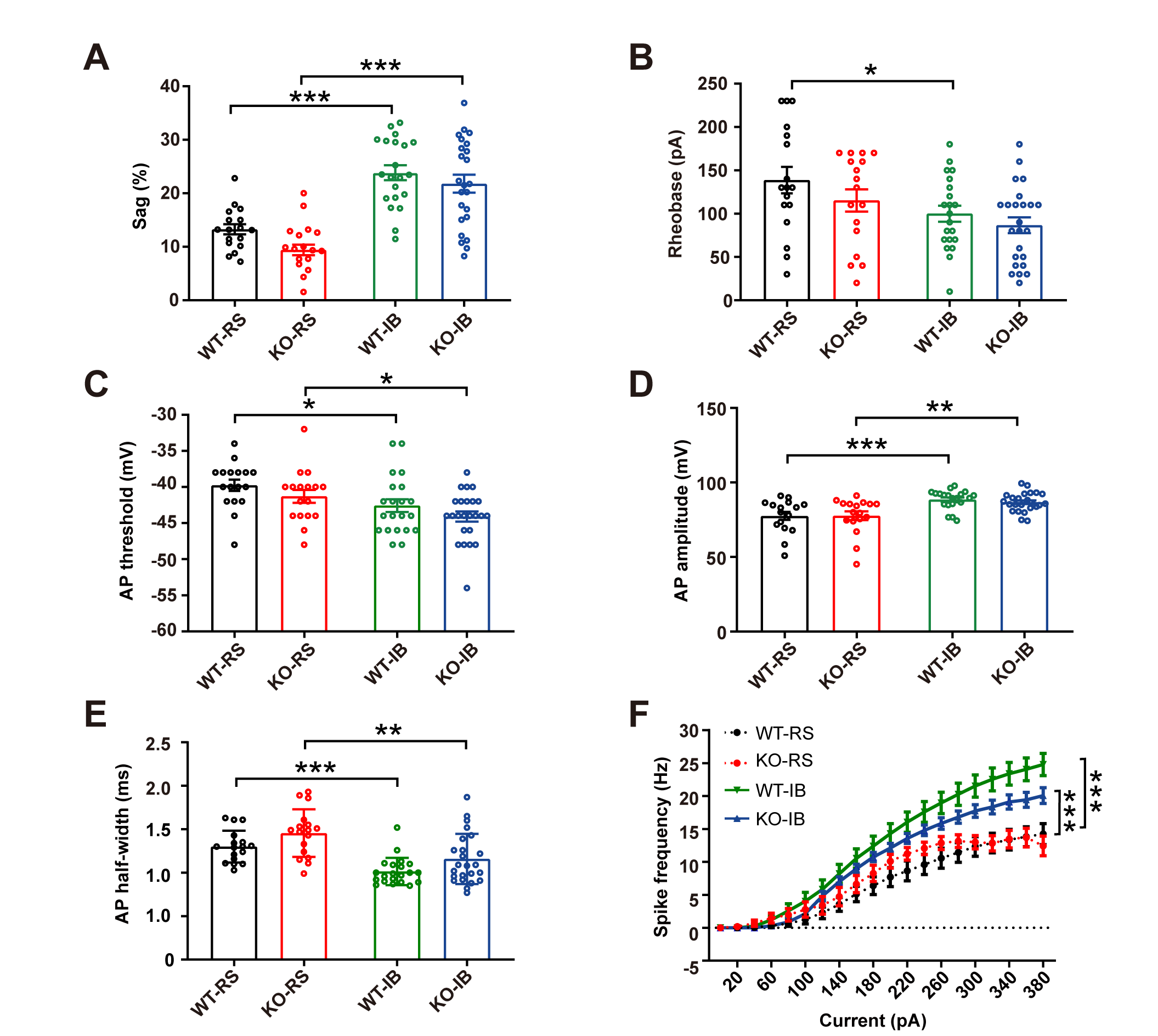

Supplement: Supplementary Figure 1 — Intrinsic electrophysiological properties of L5 pyramidal neurons of WT and KO mice. (A) Sag ratio (WT-RS vs. WT-IB, t(36) = 5.985, p < 0.0001; KO-RS vs. KO-IB, t(39) = 5.356, p < 0.0001; unpaired t-test). (B) Rheobase current (WT-RS vs. KO-RS, t(32) = 1.180, p = 0.2468; WT-IB vs. KO-IB, t(43) = 1.023, p = 0.3118; WT-RS vs. WT-IB, t(36) = 2.267, p = 0.0295; KO-RS vs. KO-IB, t(39) = 1.872, p = 0.0687; unpaired t-test). (C) AP threshold (WT-RS vs. KO-RS, t(32) = 1.290, p = 0.2064; WT-IB vs. KO-IB, t(43) = 1.349, p = 0.1844; WT-RS vs. WT-IB, t(36) = 2.317, p = 0.0263; KO-RS vs. KO-IB, t(39) = 2.479, p = 0.0176; unpaired t-test). (D) AP amplitude (WT-RS vs. KO-RS, t(32) = 0.0370, p = 0.9707; WT-IB vs. KO-IB, t(43) = 0.9384, p = 0.3533; WT-RS vs. WT-IB, t(36) = 3.856, p = 0.0005; KO-RS vs. KO-IB, t(39) = 3.119, p = 0.0034; unpaired t-test). (E) AP half-width (WT-RS vs. KO-RS, p = 0.0932; WT-IB vs. KO-IB, p = 0.1077; WT-RS vs. WT-IB, p < 0.0001; KO-RS vs. KO-IB, p < 0.0018; Mann-Whitney test). (F) I-f relationships (WT-RS vs. WT-IB, p < 0.0001; KO-RS vs. KO-IB, p < 0.0001; repeated-measures Two-way ANOVA, F(57,1120) < 0.0001). *p < 0.05; **p < 0.01; ***p < 0.001. [file Image_1.TIF]

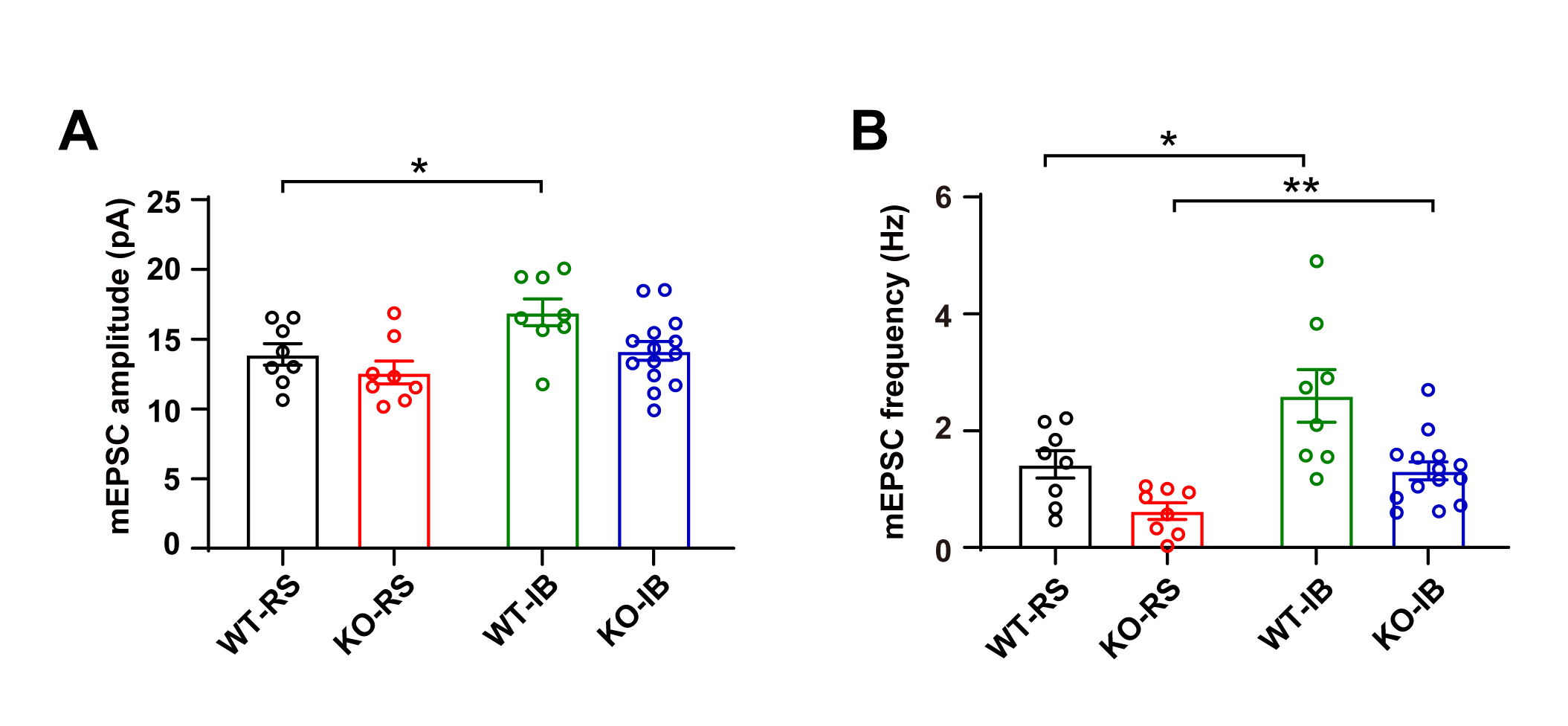

Supplement: Supplementary Figure 2 — Spontaneous glutamatergic synaptic transmission in L5 pyramidal neurons of WT and KO mice. (A) Quantification of mEPSC amplitude from RS (WT, n = 8; KO, n = 8) and IB (WT, n = 8; KO, n = 14) cells from WT (N = 7 mice) and KO mice (N = 8 mice; WT-RS vs. WT-IB, t(14) = 2.454, p = 0.0278; KO-RS vs. KO-IB, t(20) = 1.444, p = 0.1642; unpaired t-test). (B) Quantification of mEPSC frequency (WT-RS vs. WT-IB, t(14) = 2.313, p = 0.0364; KO-RS vs. KO-IB, t(20) = 2.973, p = 0.0075; unpaired t-test). *p < 0.05; **p < 0.01. [file Image_2.TIF]

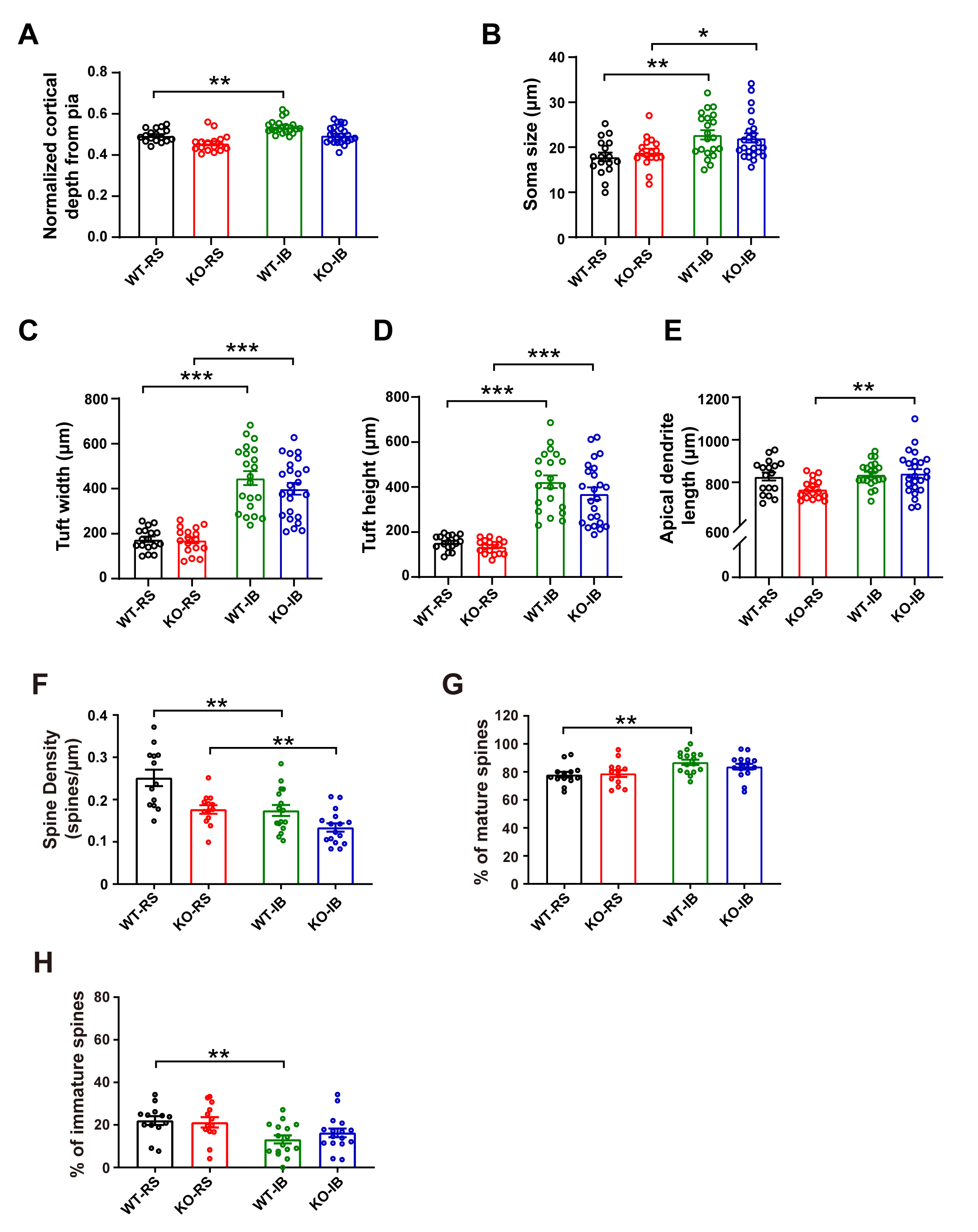

Supplement: Supplementary Figure 3 — Dendritic morphology of neurobiotin-labeled L5 pyramidal neurons. (A) Quantification of normalized cortical depth from pia (WT-RS vs. WT-IB, t(36) = 2.809, p = 0.008; KO-RS vs. KO-IB, t(39) = 1.659, p = 0.1051; unpaired t-test). (B) Quantification of soma size (WT-RS vs. WT-IB, t(36) = 3.368, p = 0.0018; KO-RS vs. KO-IB, t(39) = 2.315, p = 0.0260; unpaired t-test). (C) Quantification of tuft width (WT-RS vs. WT-IB, t(36) = 8.386, p < 0.0001; KO-RS vs. KO-IB, t(39) = 7.093, p < 0.0001; unpaired t-test). (D) Quantification of tuft height (WT-RS vs. WT-IB, t(36) = 7.560, p < 0.0001; KO-RS vs. KO-IB, t(39) = 6.932, p < 0.0001; unpaired t-test). (E) Quantification of length of primary apical dendrites (WT-RS vs. WT-IB, t(36) = 0.4417, p = 0.6614; KO-RS vs. KO-IB, t(39) = 2.872, p = 0.0066; unpaired t-test). (F) Quantification of spine density (WT-RS vs. WT-IB, t(27) = 3.394, p = 0.0021; KO-RS vs. KO-IB, t(27) = 2.977, p = 0.0061;unpaired t-test). (G) Quantification of percentages of mature spine (WT-RS vs. KO-IB, t(24) = 0.2571, p = 0.7993; WT-IB vs. KO-IB, t(30) = 1.107, p = 0.2772; WT-RS vs. WT-IB, t(27) = 3.142, p = 0.0040; KO-RS vs. KO-IB, t(27) = 1.552, p = 0.1324; unpaired t-test). (H) Quantification of percentages of immature spine (WT-RS vs. KO-RS, t(24) = 0.2571, p = 0.7993; WT-IB vs. KO-IB, t(30) = 1.107, p = 0.2772; WT-RS vs. WT-IB, t(27) = 3.142, p = 0.0040; KO-RS vs. KO-IB, t(27) = 1.552, p = 0.1324; unpaired t-test). The number of mice and neurons used in (A–E) of RS (WT, n = 17; KO, n = 17) and IB (WT, n = 21; KO, n = 24) cells from WT (N = 10 mice) and KO mice (N = 13 mice). (F–H) RS (WT, n = 13; KO, n = 13) and IB (WT, n = 16; KO, n = 16) cells in WT (N = 6) and KO mice (N = 9). *p < 0.05; **p < 0.01; ***p < 0.001. [file Image_3.TIF]

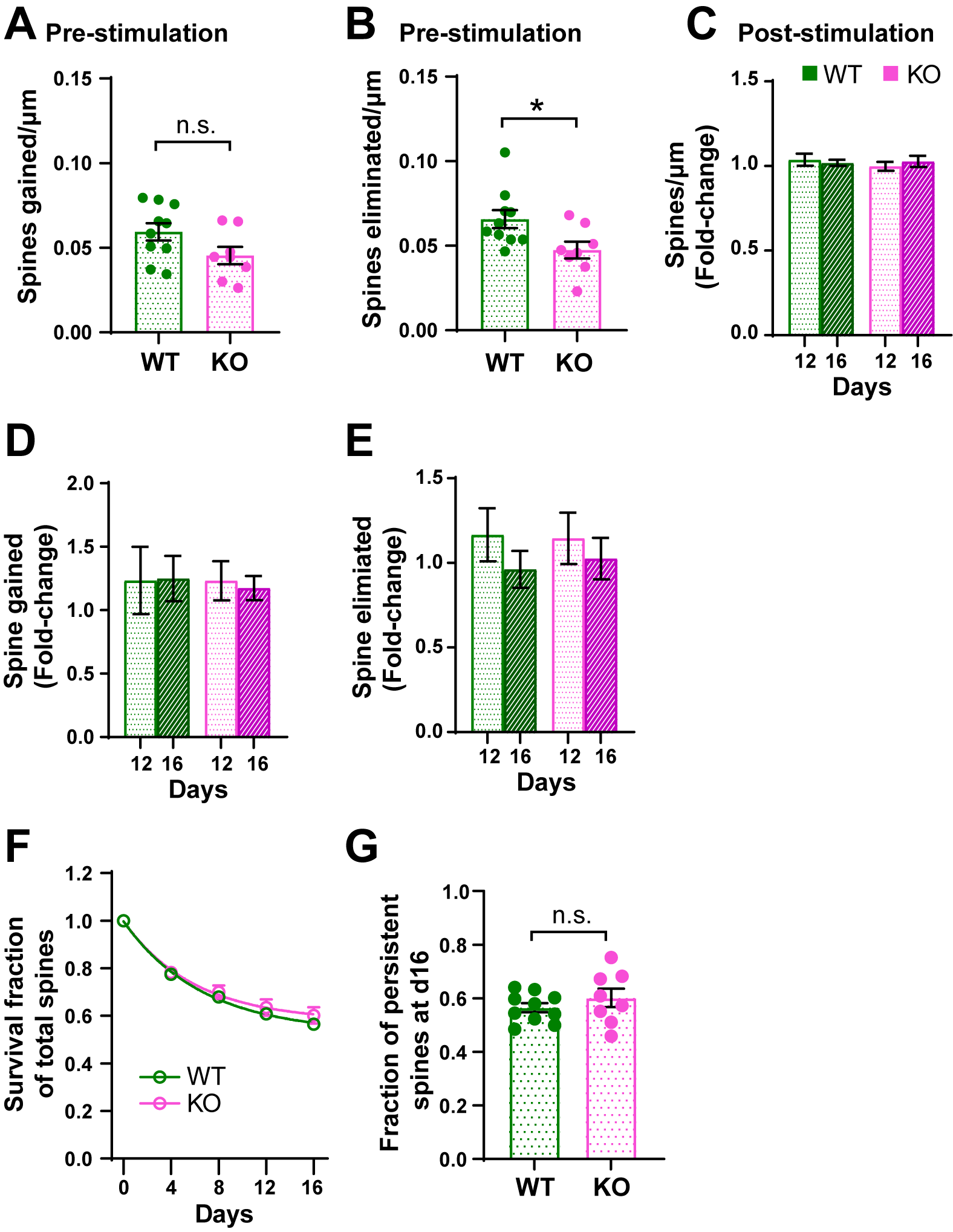

Supplement: Supplementary Figure 4 — Chronic in vivo quantification of density and dynamics of dendritic spines of L5 pyramidal neurons in S1BF of WT and p53KO mice before and after stimulation. (A,B) Pre-stimulation dendritic spine gained and eliminated ratios. (C–E) Post-stimulation (pre-stim. vs. d12 and d16) fold-change of dendritic spine density (C), gained spines (D), and eliminated spines (E). (F) Survival function of all the spines present at d0. (G) Fraction of persistent spines at d16. *p < 0.05. [file Image_4.TIF]
